# Supplementary material for: Synthesising a minimal cell with artificial metabolic pathways
Source: Commun Chem. 2023 Mar 28;6:56. doi: 10.1038/s42004-023-00856-y (PMC10050237; doi:10.1038/s42004-023-00856-y)
Supplement: Supplementary file 2 — Supplementary Information [file 42004_2023_856_MOESM2_ESM.pdf]

Supplementary Information

Synthesising a minimal cell with artificial metabolic pathways

Minoru Kurisu[1], Ryosuke Katayama[1], Yuka Sakuma[1], Toshihiro Kawakatsu[1],
Peter Walde[2], and Masayuki Imai[1*]

[1]Department of Physics, Graduate School of Science, Tohoku University, 6-3 Aoba, Aramaki, Aoba, Sendai 980-8578, Japan. *email: imai@bio.phys.tohoku.ac.jp
[2]Department of Materials, ETH Zürich, Vladmir-Prelog-Weg 5, CH-8093 Zürich, Switzerland

Contents

Supplementary Note 1.
Chemical scheme of artificial metabolic pathway *4*

1-1. Reaction pathway in “energy production” unit *4*

Supplementary Scheme 1.

1-2. Reaction pathway in “information molecule synthesis” unit *5*

Supplementary Scheme 2.

1-3. Pathway in “membrane growth” unit *8*

Supplementary Figure 1.

Supplementary Note 2.
Installing the “energy production” unit in the artificial metabolic pathway *10*

2-1. PANI-ES synthesis by the direct supply of H_2O_2: *Control experiment-0* *10*

Supplementary Figure 2-1.

2-2. AOT membrane growth coupled with PANI-ES synthesis by the direct supply of H_2O_2: *Control experiment-1* *13*

Supplementary Figure 2-2.

Supplementary Note 3.
Kinetic model of artificial metabolic pathway *16*

3-1. Reduced model reaction scheme for artificial metabolic pathways *16*

Supplementary Figure 3-1.

3-2. Rate equations of reduced model reactions *20*

3-3. Simultaneous differential equations describing the kinetics of artificial metabolic pathways *22*

(i) Kinetic model for full artificial metabolic pathways: *Model-1*

(ii) Kinetic model for PANI-ES synthesis in cascade reaction system: *Model-2*

(iii) Kinetic model for PANI-ES synthesis without “energy production” unit: *Model-3*

(iv) Kinetic model for membrane growth of AOT GUV coupled with PANI-ES synthesis without “energy production” unit: *Model-4*

Supplementary Figure 3-2.

3-4. Parameter list of the kinetic model *28*

Supplementary Table 1.

Supplementary Note 4.
Time dependence of ingredient (D-glucose and aniline) concentrations during the progress of artificial metabolic reactions: experiment vs kinetic model *31*
Supplementary Figure 4.

Supplementary Note 5.
Osmotic inflation of AOT vesicles *33*
Supplementary Figure 5.

Supplementary Note 6.
Size distribution of AOT + Chol daughter vesicles after each division *37*
Supplementary Figure 6.

Supplementary Note 7.
Enthalpy change due to the interaction between AOT and PANI-ES *39*
Supplementary Figure 7.

Supplementary Note 8.
Deformation and division of vesicle induced by inverse cone-shaped amphiphile...*41*
8-1. Theoretical analysis for the deformation of binary AOT + Chol vesicles *41*
8-2. Growth and division of binary AOT + phosphatidylethanolamine (PE) vesicles *42*
Supplementary Figure 8.

Supplementary Note 9.
Descriptions on supporting movies *45*
Supplementary Movie 1.
Supplementary Movie 2.
Supplementary Movie 3.
Supplementary Movie 4, 5, and **6.**

Supplementary Note 10.
Supplementary References *47*

Supplementary Note 1.

Chemical scheme of the artificial metabolic pathway.

1-1. Reaction pathway in "energy production" unit ((R1) in Fig. 1b orange)

The reaction pathway that produces H_2O_2, energy currency in the artificial metabolic pathway, is shown in **Supplementary Scheme 1**. In 20 mM NaH_2PO_4 solution (pH = 4.3), H_2O_2 is produced by the oxidation of D-glucose with dissolved O_2 and glucose oxidase (GOD) catalysis (R1). More specifically, the GOD-catalysed production of H_2O_2 is described as two enzymatic reactions and subsequent hydrolysis of by-product as follows[1]: GOD in which its cofactor flavin adenine dinucleotide (FAD) is in the oxidised form (GOD-FAD) firstly reacts with one β-D-glucose molecule to yield one D-glucono-δ-lactone molecule and GOD in which FAD is in the reduced form (GOD-$FADH_2$) (R1a). GOD-$FADH_2$ reacts with one dissolved O_2 molecule to produce one H_2O_2 molecule and GOD-FAD (R1b). The main product, D-glucono-δ-lactone, is hydrolysed to D-gluconic acid (R1c). Overall, one dissolved O_2 molecule is reduced to one H_2O_2 molecule as a by-product (R1) in a GOD cycle, while one β-D-glucose molecule is oxidised to form D-gluconic acid.

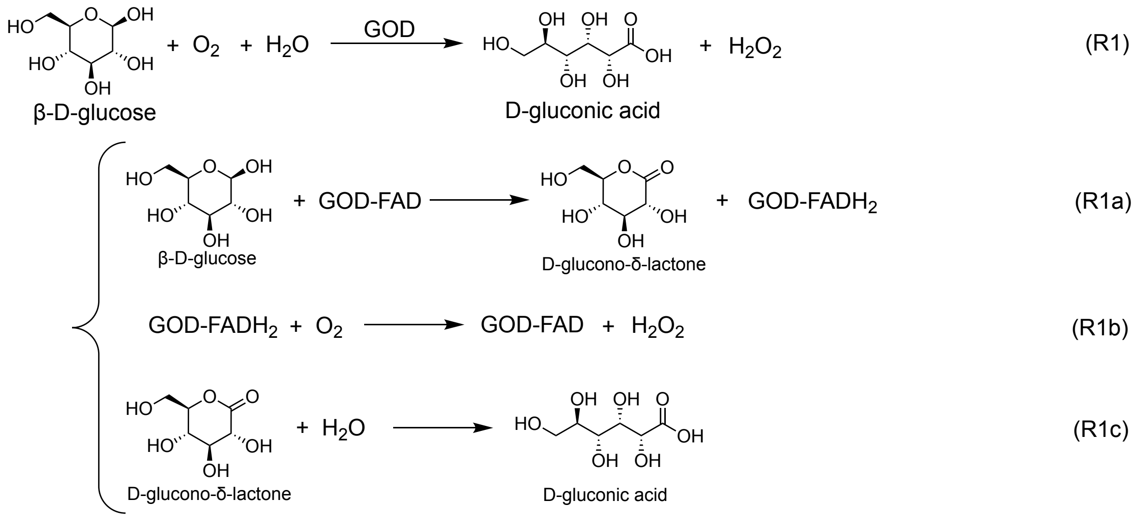

Supplementary Scheme 1. Reaction scheme of "energy production" unit, corresponding to (R1) in Fig. 1b orange.

The GOD-catalysed production of H_2O_2 (R1) is described as a GOD cycle to produce a by-product H_2O_2 (R1a and R1b) and subsequent hydrolysis of main product, D-glucono-δ-lactone (R1c). Typically, the initial reaction condition involves 100 mM D-glucose, 1.0 μM GOD, and dissolved O_2 in 20 mM NaH_2PO_4 solution (pH = 4.3) with other reaction components for PANI-ES synthesis (see **Methods**).

1-2. Reaction pathway in "information molecule synthesis" unit ((R2–R8) in Fig. 1b green)

Plausible reaction pathways for the synthesis of polyaniline in its emeraldine salt form (PANI-ES) from aniline with HRPC and H_2O_2 are shown in **Supplementary Scheme 2**[2–4]. In the presence of AOT vesicles in NaH_2PO_4 solution (pH = 4.3), HRPC molecules and aniline molecules are extensively bound to the AOT vesicle surface via electrostatic interaction and/or hydrogen bonding[5–8]. Therefore, the following reaction steps are considered to be localised on the AOT vesicle surface: The HRPC/H_2O_2-catalysed oxidation of aniline leads to the production of the anilino radical (R2), which is represented as the peroxidase cycle of heme (haem) peroxidase involving native Fe(III) peroxidase, intermediate compound I with a Fe(IV) oxoferryl species with a cation radical, and compound II with a Fe(IV) oxoferryl species[9,10] (R2a, R2b, and R2c). The anilino radical is protonated to the aniline radical cation (R3). While the unpaired electron of aniline radical cation can be localised either on the carbon atom in *para*-position (P), in one of the two *ortho*-positions (O1 and O2), or on the nitrogen atom (N), the aniline radical cation having the *para*-position radical (P) reacts preferentially with that having the nitrogen position radical (N) in the presence of template AOT vesicle to form the *para*-NC-coupled aniline dimer (*p*-aminodiphenylamine, PADPA) (R4) due to the specific hydrogen bonding[4,11,12] (see **Supplementary Note 7**). It should be noted that the AOT molecules play a role as the counter anions ($A^{\ominus}$) to restrict the polymerisation process on their vesicle surface as the "template"[13,14]. Oxidation of PADPA with H_2O_2 yields the aniline dimer (*N*-phenyl-1,4-benzequinonediimine, PBQ) in its dication form, followed by the formation of its mesomeric structure, the aniline dimer diradical dication form (R5). Then, PBQ reacts with an aniline radical cation having the *para*-position radical (P) in the presence of template AOT vesicle to form aniline trimer radical cation (R6). After the trimer radical cation is partially oxidised with H_2O_2 (R7), it reacts with the aniline radical cation having the nitrogen position radical (N) in the presence of template AOT vesicle to form a half-oxidised aniline tetramer dication that is a repeating unit of PANI-ES (R8). The overall stoichiometry of the reaction to obtain one repeating unit of PANI-ES is given as (R_{PANI}) in **Supplementary Scheme 2**[4]. It should be noted note that the formation of PANI-ES (R2 – R8) coupled with the in-situ formation of H_2O_2 ((R1) in **Supplementary Scheme 1**) was confirmed in our previous work using 100 mM NaH_2PO_4 solution (pH = 4.3) instead of the present condition, 20 mM NaH_2PO_4 solution (pH = 4.3)[15]. The two-dimensional micro-Raman mapping shows that the obtained PANI-ES is distributed nearly homogeneously on the AOT GUV surface (see **Fig. 2d**). The key feature in the reactions is that the propagation of a PANI-ES chain is realised by adding one aniline radical cation (S^*) having para- or nitrogen position radical (P or N, respectively) to the oxidised radical cation of the growth end of a PANI-ES chain (${P_n}^*$).

HRPC: horseradish peroxidase isoenzyme C (enzyme)
$A^{\ominus}$: counter AOT anion (vesicle surface)

Oxidation of aniline catalysed by HRPC:

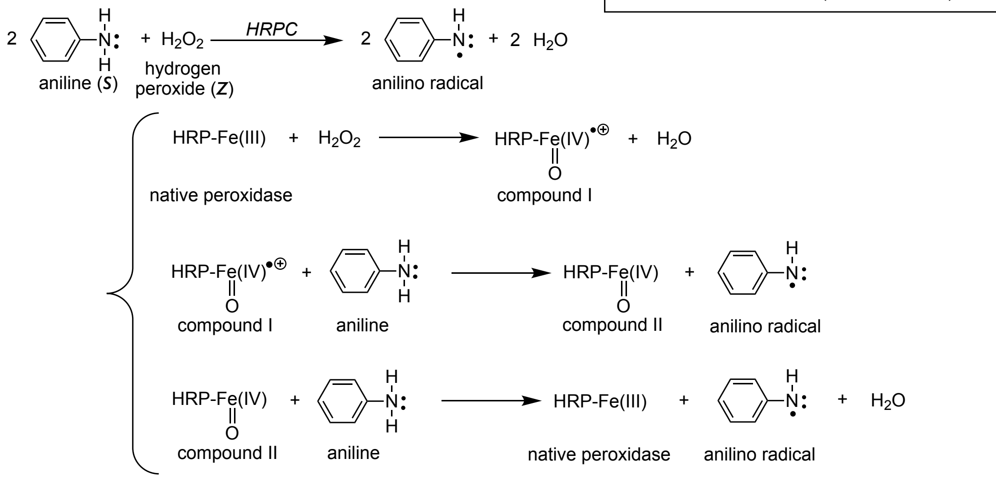

(R2)

(R2a)

(R2b)

(R2c)

Protonation of anilno radical:

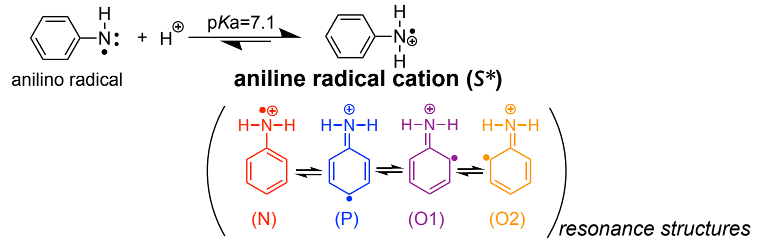

(R3)

Formation of aniline dimer (PADPA):

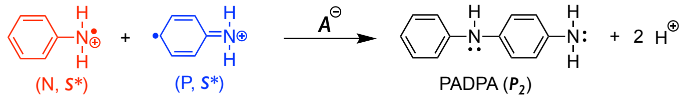

(R4)

Oxidation of PADPA with H_2O_2:

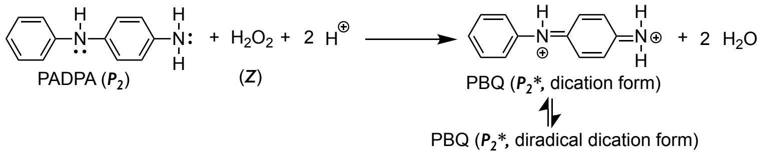

(R5)

Formation of aniline trimer radical cation:

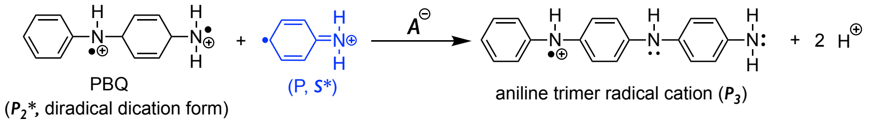

(R6)

Partial oxidation of aniline trimer radical cation:

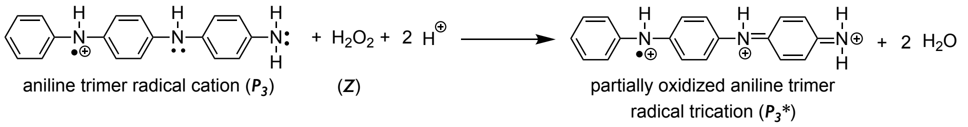

(R7)

Formation of half-oxidised and half-reduced tetraaniline (repeating unit of PANI-ES):

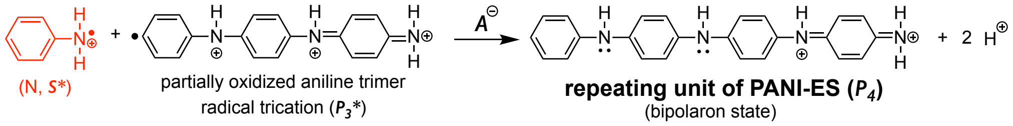

(R8)

Overall net reaction for the formation of PANI-ES repeating unit:

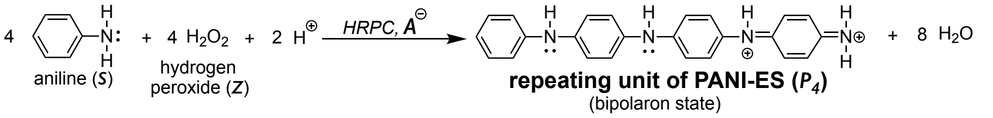

(R_{PANI})

Supplementary Scheme 2. Plausible reaction scheme of the "information molecule synthesis" unit, corresponding to (R2–R8) in Fig. 1b green.

The HRPC-catalysed formation of PANI-ES on the surface of AOT vesicle membranes is described as (R2) – (R8). The HRPC-catalysed oxidation of aniline with H_2O_2 (R2) consists of a peroxidase cycle with a native peroxidase and two intermediate compounds (R2a), (R2b), and (R2c). Typically, the initial reaction condition involves 3.0 mM AOT (vesicles), 4.0 mM aniline, 0.92 μM HRPC in 20 mM NaH_2PO_4 solution (pH = 4.3) with other reaction components for the GOD-catalysed H_2O_2 production (see **Supplementary Scheme 1**), such as 100 mM D-glucose and 1.0 μM GOD. The symbols shown for each substance (*e.g.*,S, Z and P_n) are used in the model reaction schemes in **Supplementary Fig. 3-1** and **3-2**.

1-3. Pathway in "membrane growth" unit ((R9) in Fig. 1b blue)

PANI-ES on the surface of AOT GUVs significantly enhances membrane growth (**Fig. 3a** and red circles in **Fig. 3b**), which indicates transport of AOT molecules in the external solution to the vesicle membrane through specific interactions with PANI-ES. Supposed molecular mechanisms of AOT vesicle membrane growth in the presence of PANI-ES are schematically shown in **Supplementary Fig. 1**.

$$A_{\mathrm{m}} + P_n \rightleftarrows (A_{\mathrm{m}}P_n) \rightleftarrows (P_nA_{\mathrm{v}}^{\mathrm{o}}) \rightarrow P_n + A_{\mathrm{v}}^{\mathrm{o}} \qquad \text{(R9)}$$

Here "A_{m}" represents an AOT molecule monodispersed in the external solution, "$A_{\mathrm{v}}^{\mathrm{o}}$" represents an AOT molecule in the outer leaflet of the bilayer membrane, and "P_n" represents PANI-ES on AOT vesicle membranes. The binding of AOT molecules (A_{m}) to the surface-confined PANI-ES (P_n), ($A_{\mathrm{m}}P_n$), through specific interactions between PANI-ES and a head group of an AOT (see also **Supplementary Note 7**)[4,11,12,16] will decrease the hydrophilicity of AOT molecule. Then, the bound AOT molecules prefer to incorporate into the outer leaflet of vesicle membranes ($P_nA_{\mathrm{v}}^{\mathrm{o}}$). AOT molecules incorporated in the outer leaflet, $A_{\mathrm{v}}^{\mathrm{o}}$, are transported to the inner leaflet due by the fast flip-flop motion to relax the excess tension in the outer leaflet[17–19], which results in the rapid growth of the vesicle membrane.

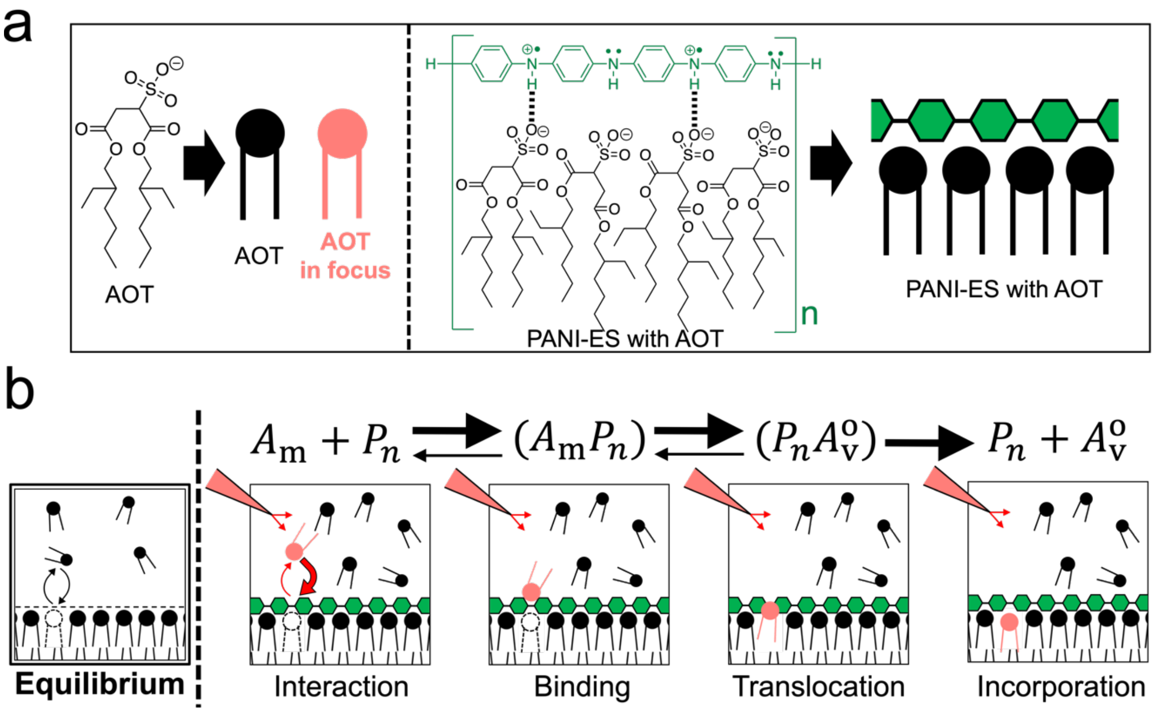

Supplementary Figure 1. Supposed molecular mechanism of membrane growth of AOT GUVs coupled with surface-confined PANI-ES formation.

a, Schematic representations of AOT molecules and PANI-ES synthesised on AOT membrane.

b, Supposed molecular mechanism of AOT membrane growth coupled with PANI-ES on the surface of AOT GUVs (**Fig. 3a** and red circles in **Fig. 3b**). An AOT molecule in the external solution that binds to PANI-ES through the specific hydrogen bonding is highlighted in red. A red pipette at the upper left corner in each figure indicates micro-injection of an aqueous solution of AOT micelles. The growth mechanism is explained in the text.

Supplementary Note 2.

Installing the "energy production" unit in the artificial metabolic pathway.

In the artificial metabolic pathway, the information polymer, PANI-ES, is enzymatically synthesised in the presence of template AOT vesicles, where the reaction trigger H_2O_2 is in-situ produced by the GOD-catalysed reaction system (energy production unit)[15] ((R1) – (R8) in **Fig. 1b** and **Supplementary Note 1-1**). To examine the effect of the energy production unit on the synthesis of PANI-ES and the membrane growth, we synthesised PANI-ES using externally supplied H_2O_2 ((R2) – (R9) in **Fig. 1b**, **Supplementary Note 1-2**, and **1-3**)[4,20]. These types of experiments are referred to as control experiments. Based on the control experiments, we determined the rate constants that appeared in the kinetic model (Supplementary Eq.(V1) – (V5) in **Supplementary Note 3-2**) of the artificial metabolic pathway.

2-1. PANI-ES synthesis by the direct supply of H_2O_2: *Control experiment-0*

Here the reaction condition for the HRPC-catalysed polymerisation of aniline in the presence of AOT LUVs triggered by the direct addition of H_2O_2 (control experiment-0) is the same as the one we developed previously (**Supplementary Fig. 2-1a**)[20]. All components of the reaction mixture, except for the H_2O_2 solution to trigger the polymerisation, were added to 349.9 μL of 20 mM NaH_2PO_4 solution (pH = 4.3) in a 5 mL Eppendorf polypropylene tube: 75 μL AOT LUV suspension (20 mM AOT in 20 mM NaH_2PO_4 solution), 50 μL aniline solution (40 mM in 20 mM NaH_2PO_4 solution, pH adjusted to 4.3 with H_3PO_4), and 25 μL HRPC solution (18.4 μM in 20 mM NaH_2PO_4 solution, pH = 4.3). After gentle mixing, the reaction was triggered by the quick addition of 1.13 μL of freshly prepared H_2O_2 solution (2.0 M in water). The initial concentration in the reaction mixture was as follows: 3.0 mM AOT (LUVs), 4.0 mM aniline, 0.92 μM HRPC, 4.5 mM H_2O_2, and 20 mM NaH_2PO_4 (pH = 4.3) in a reaction volume of 0.50 mL at $T \sim 25^{\circ}C$ (room temperature). It should be noted that no AOT micelles were supplied in this experiment (*i.e.*, no membrane growth), which is called control experiment-0.

The UV/Vis/NIR absorption spectra of PANI-ES obtained by the control experiment-0 (blue dashed line) and the cascade reaction experiment (red solid line) are shown in **Supplementary Fig. 2-1b**, where absorption spectra were recorded after 24 h from the start of the reaction. Both spectra show similar profiles with maximum absorbance at $\lambda \sim 300$, ~420, and ~1000 nm, which is indicative of $\pi \rightarrow \pi^*$, polaron $\rightarrow \pi^*$, and $\pi \rightarrow$ polaron transition of PANI-ES in the polaron state with its unpaired electrons, respectively[21–24]. The progress of PANI-ES synthesis in the control experiment-0 was monitored by the time evolution of an absorption peak at $\lambda = 1000$ nm. The absorbance at $\lambda = 1000$ nm, $a_{1000}(t)$, was normalised by the absorbance recorded after 24 h

from the start of the reaction, a_{1000} (24 h), (blue line in **Supplementary Fig. 2-1c**). The synthesis of PANI-ES in the control experiment-0 proceeded rapidly in the first 70 sec and then gradually slowed down, which agrees with the time scale of the cascade reaction experiment (**Fig. 2e**). Thus, no significant difference in the synthesis of PANI-ES between the cascade reaction scheme and the direct H_2O_2 supply scheme, *i.e.*, the energy production unit was installed successfully in terms of PANI-ES synthesis.

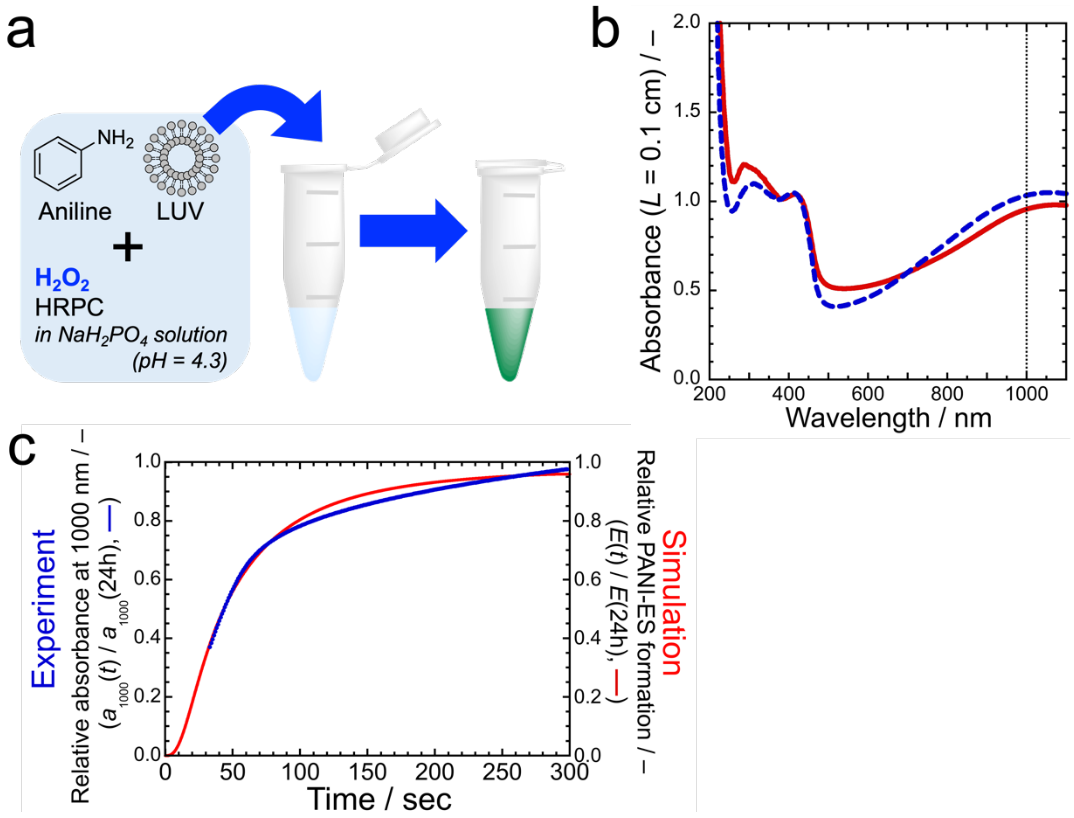

Supplementary Figure 2-1. PANI-ES synthesis by the direct supply of H_2O_2: Control experiment-0 (without energy production unit).

a, Schematic procedure of the control experiment-0, PANI-ES synthesis by HRPC-catalysed system in the presence of AOT LUVs triggered by the addition of H_2O_2, (R2) – (R8). See text for the detailed reaction procedure. After quick addition of the H_2O_2 solution and gentle mixing, the initially colourless reaction mixture soon turned dark blue and then dark green.

b, UV/Vis/NIR absorption spectra of the polymerisation products obtained either by the GOD/HRPC-catalysed cascade reaction, (R1) – (R8), (red solid line) or by the direct supply of H_2O_2 (control experiment-0), (R2) – (R8), (blue dashed line). Both spectra were recorded after 24 h from the start of the reactions at $T \sim 25^\circ$C. The black dotted line represents the absorbance at λ = 1000 nm.

c, Time dependence of relative absorbance at λ = 1000 nm of the reaction mixture (blue line) in the control experiment-0. The growth profile, a_{1000} (t), is normalised by the absorbance recorded after 24 h from the start of the reaction, a_{1000} (24h), of the blue dashed line in **b**. The absorbance at λ = 1000 nm of the reaction mixture was recorded after 30 s from the addition of H_2O_2 due to the sample preparation, with the time step of 1.0 s. The red line is the fitting curve based on the kinetic model-3, which gives the rate constants k_3 and k_4 (**Supplementary Note 3-3(iii)**).

2-2. AOT membrane growth coupled with PANI-ES synthesis by the direct supply of H_2O_2: *Control experiment-1*

We observed that the membrane growth of AOT GUV is promoted by the surface-confined PANI-ES synthesis in the cascade reaction system, (R1) – (R9) (**Fig. 3a** and **b**). To examine the effect of the energy production unit on the kinetics of AOT membrane growth, here we performed a control experiment on AOT membrane growth coupled with the supply of AOT micelles and the PANI-ES synthesis, where the energy currency H_2O_2 was directly supplied (control experiment-1), *i.e.*, without the energy production unit, (R2) – (R9). In the control experiment-1, the double micro-injection setup was adopted, which is almost the same experimental system as the AOT GUV growth involving the cascade reactions (see **Methods**), except for the compositions of the bulk and micro-injected solutions. A schematic of the double micro-injection setup for the control experiment-1 is shown in **Supplementary Fig. 2-2a**. The detailed experimental procedure was as follows: First, 2.0 mL of AOT GUV suspension mixed with the reaction components for HRPC-catalysed polymerisation, except for H_2O_2, was carefully transferred from a microtube into a glass-bottom sample chamber. The concentrations of each component of this reaction mixture were as follows: 3.0 mM AOT (as GUVs; critical vesiculation concentration, cvc, ~1.5 mM), 4.0 mM (2.0 mM or 1.0 mM in reference experiments) aniline, and 0.92 μM HRPC in 20 mM NaH_2PO_4 solution (pH = 4.3). Then, the micro-pipettes were set for the target GUV located at the bottom of the chamber. The polymerisation of aniline was triggered by micro-injecting a freshly prepared 20 mM AOT micellar solution containing 2.0 M H_2O_2. Simultaneously, the same solution as the bulk solution except for AOT GUVs was micro-injected from the other micro-pipette as a counter flow to prevent floating away of the target GUV (**Supplementary Fig. 2-2a)**: 4.0 mM (2.0 mM or 1.0 mM in reference experiments) aniline and 0.92 μM HRPC in 20 mM NaH_2PO_4 solution (pH = 4.3). Both micro-injection solutions were pressed through a 0.2 μm polypropylene filter before use. The distance between the tips of the two micro-pipettes was ~100 μm, and the distance from the tip of the micro-pipettes to the bottom of the chamber was ~40 μm. The injection pressure was ~70 hPa, corresponding to an injection flow rate of ~0.1 nL s^{-1}. The two injection flows trapped the target GUV at an almost fixed point at the bottom of the chamber during the reaction. The morphological changes of the target AOT GUVs were observed by the phase-contrast light microscope (see **Methods**).

In response to the micro-injection of AOT micelles and H_2O_2, the target AOT GUVs showed membrane growth by incorporating AOT molecules from the external solution. The phase-contrast micrographs of AOT GUV membrane growth and the corresponding time evolution of the surface area normalised by the initial surface area of the target GUV, $A(t)/A(0)$, are shown in **Supplementary Fig. 2-2b** (concentration of aniline; 4 mM) and **2-2c** [concentration of aniline; 4 mM (red circles), 2 mM (blue squares) and 1 mM (green triangles)], respectively. The vesicle

surface area was estimated by approximating the vesicle shape with an axisymmetric prolate shape. When the micro-injection was started, the initially spherical AOT GUVs began to grow into a prolate shape after ~20 sec, which coincides with the timescale of PANI-ES formation in the control experiment-0 (**Supplementary Fig. 2-1c**). This time scale was faster than the timescale observed in the optimised cascade reaction system (~50 s) (**Fig. 3a** and **b**). It should be noted that the concentration of supplied H_2O_2 was different between control experiment-0 (4.5 mM in bulk solution, optimal initial concentration for the PANI-ES synthesis[4]) and control experiment-1 (2.0 M from a micro-pipette, the same setup as our previous work[20]). The AOT GUVs showed exponential membrane growth coupled with the surface-confined synthesis of PANI-ES. Except for the growth timescale, there was no significant difference in the growth of AOT GUV between the cascade reaction scheme and the direct H_2O_2 supply scheme.

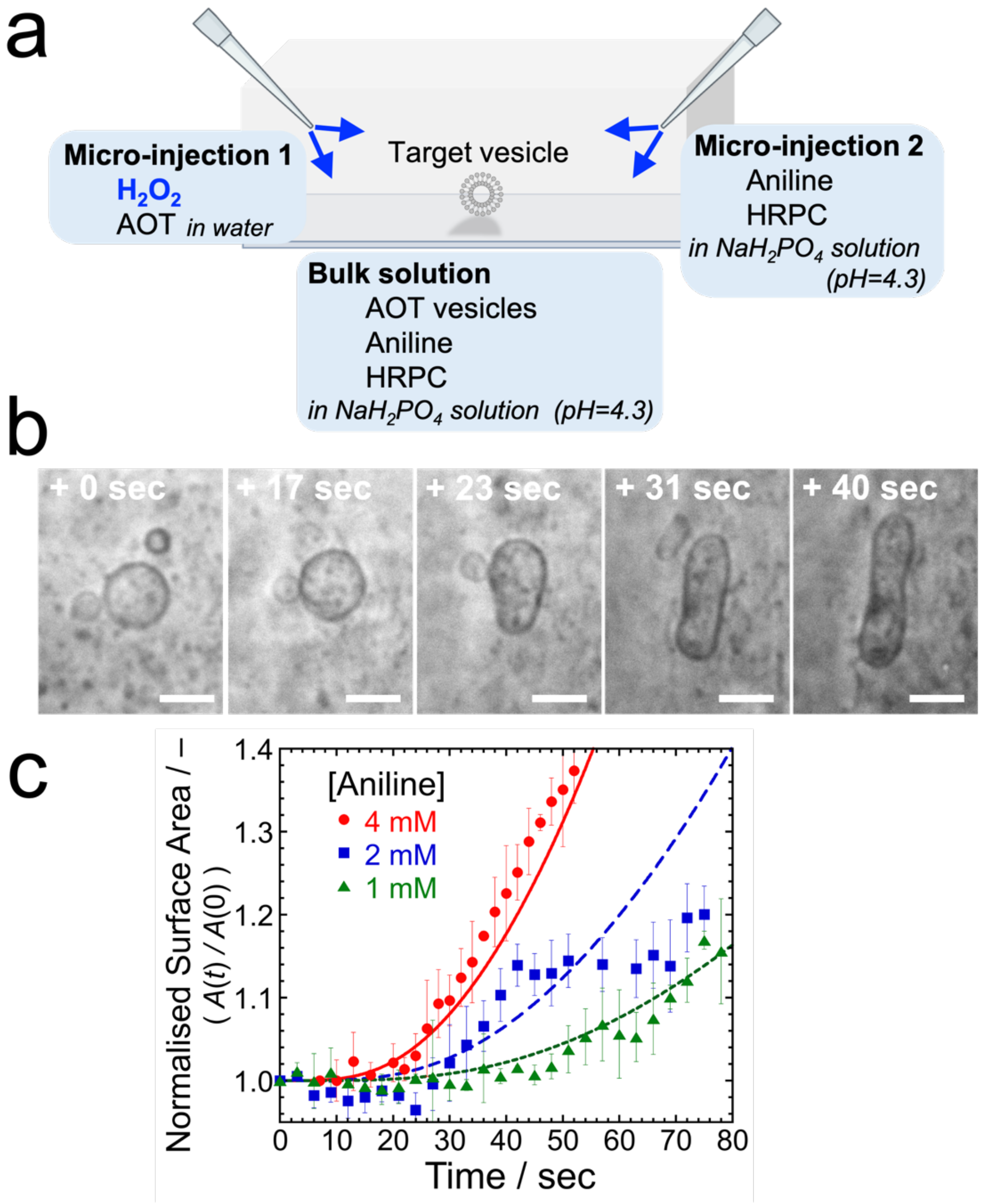

Supplementary Figure 2-2. Membrane growth of AOT GUVs coupled with PANI-ES synthesis triggered by direct H_2O_2 supply.

a, Schematic of double micro-injection setup for membrane growth of AOT GUVs coupled with PANI-ES synthesis triggered by the direct H_2O_2 supply, control experiment-1. Micro-injection 1: 2.0 M H_2O_2 and 20 mM AOT (micelles). Micro-injection 2: 4.0 mM aniline (2.0 mM or 1.0 mM in reference experiments), 0.92 μM HRPC and 20 mM NaH_2PO_4 (pH = 4.3). Bulk solution: 3.0 mM AOT (GUVs), 4.0 mM aniline (2.0 mM or 1.0 mM in reference experiments), 0.92 μM HRPC and 20 mM NaH_2PO_4 (pH = 4.3). The distance between the tips of two micro-pipettes: ~100 μm. From the bottom of the chamber to the tips of micro-pipettes: ~40 μm. Injection pressure: ~70 hPa (injection flow rate of ~0.1 nL s^{-1}).

b, Phase-contrast micrographs of growing AOT GUV in the control experiment-1. The concentration of aniline in the reaction system was 4.0 mM. The elapsed time after starting the micro-injection is indicated in each image. Length of the scale bars: 10 μm.

c, Growth of AOT GUVs in the control experiment-1. The time evolution of membrane surface area of GUVs normalised by their initial surface area, $A(t)/A(0)$, is plotted for each aniline concentration (red circles: 4.0 mM, blue squares: 2.0 mM, and green triangles: 1.0 mM). The error bars indicate standard deviations estimated from three to six different experiments. The experimentally observed membrane growth was reproduced with the kinetic model-4, which gives the rate constants k_5 (**Supplementary Note 3-3(iv)**), (aniline concentration; red solid line: 4.0 mM, blue dashed line: 2.0 mM, and green dotted line: 1.0 mM).

Supplementary Note 3.

Kinetic model of artificial metabolic pathway

A living system is maintained by a very complex network of spatially and timely controlled chemical transformations. A quantitative description of such a chemical reaction network is significant for understanding living systems. However, since the metabolic pathways are so complex that even the simplest bacterium has around 250 essential genes, it is an extremely difficult work[25,26]. One of the great advantages of the synthetic minimal cell study is that it might provide a quantitative understanding of the chemical network. Our artificial metabolic pathways are composed of energy production (R1), synthesis of information polymer, (R2) – (R8), and membrane growth (R9) units, as shown in **Fig. 1b** and **Supplementary Note 1**. Since these chemical pathways are well characterised, we developed a kinetic model of our artificial metabolic pathway to reveal the coupling among three essential chemical units.

3-1. Reduced model reaction scheme for artificial metabolic pathways

The model reaction scheme of our artificial metabolic pathways, reaction (M1) – (M5), are expressed by,

$$R_\mathrm{a} + R_\mathrm{b} + X \xrightarrow{v_1} Z + X \tag{M1}$$

$$2\,S + Y + Z \xrightarrow{v_2} 2\,S^* + Y \tag{M2}$$

$$2\,S^* + 2A_\mathrm{v}^\mathrm{o} \xrightarrow{v_3} P_2 + 2A_\mathrm{v}^\mathrm{o} \tag{M3}$$

$$S^* + P_n + Z + 2A_\mathrm{v}^\mathrm{o} \xrightarrow{v_4} P_{n+1} + 2A_\mathrm{v}^\mathrm{o} \tag{M4}$$

$$A_\mathrm{m} + P_n \xrightarrow{v_5} A_\mathrm{v}^\mathrm{o} + P_n\,, \tag{M5}$$

which reduces the full reaction scheme, (R1) – (R9) in **Fig. 1b** (**Supplementary Fig. 3-1a**). The details of each reduced reaction are as follows. Energy production reaction, reaction (M1), **(Supplementary Fig. 3-1b**): The model reaction (M1) is based on the actual reaction (R1) in **Fig. 1b** and **Supplementary Scheme 1**. The GOD (X) -catalysed oxidation of D-glucose (R_a) using dissolved oxygen (R_b) produces H_2O_2 (Z), the energy currency in the artificial metabolic pathways. Here the native enzyme X is activated to X^* by using R_a and by forming a D-glucono-δ-lactone. Then, the activated enzyme X^* produces energy currency Z from R_b and returns to the native state X **(Supplementary Fig. 3-1b**). This kinetics strictly obeys the two-substrate ping-pong mechanism[27,28]. Production of active monomer, reaction (M2) (**Supplementary Fig. 3-1c**): The HRPC (Y)/H_2O_2 (Z) -catalysed oxidation of aniline (S) to anilino radical (*not shown*), which is followed by the protonation to aniline radical cation (S^*), is described based on (R2) and (R3). Here the anilino radical is assumed to be instantly protonated to aniline radical cation due to its high pK_a value (pK_a = 7.1)[5,29]. The native enzyme Y receives energy currency Z, then becomes the activated state Y^*. The activated enzyme Y^* reacts with

two monomers S to produce two activated monomers S^*, while Y^* transforms its own states to another activation state Y^{**} and then to the native state Y (**Supplementary Fig. 3-1c**). This kinetics obeys the irreversible ping-pong mechanism in which intermediate complexes between substrates and enzymes are very short-lived[30]. Initiation reaction of information polymer synthesis, reaction (M3), (**Supplementary Fig. 3-1d**): The initiation reaction is described based on (R4) as the reaction between two aniline radical cations (S^*) to form aniline dimer (P_2) on the surface of an AOT vesicle membrane ($A_{\mathrm{v}}^{\mathrm{o}}$). According to both experiment and simulation, aniline and aniline radical cation molecules are expected to be adsorbed to the surface of the negatively charged AOT vesicle membrane due to electrostatic interaction and/or hydrogen bonding involving an amino group of aniline and a sulfonate head group of AOT[5–8], which results in the production of the *para*-NC-coupled aniline dimer. Here the dimerisation of aniline radical cations is described as the Langmuir-Hinshelwood mechanism (**Supplementary Fig. 3-1d**), where K_{S^*} is the adsorption equilibrium constant of aniline radical cation, and "(a)" represents the adsorbed state. Propagation reaction of information polymer synthesis, reaction (M4), **(Supplementary Fig. 3-1e**): The propagation reaction is described by the combination of two reaction steps on AOT membranes based on (R5) – (R8), where PANI-ES chain (P_n: $n \geq 2$) is oxidised with H_2O_2 (Z) to produce the oxidised PANI-ES chain with the degree of polymerisation *n* (P_n^*), and then aniline radical cation molecules (S^*) react with P_n^* to produce elongated PANI-ES chain (P_{n+1}). This reaction kinetics is described as the irreversible ping-pong mechanism combined with the Langmuir-Hinshelwood mechanism **(Supplementary Fig. 3-1e**). Here we assumed that the coverage of the vesicle surface by PANI-ES chains does not affect the kinetics of any surface-confined reaction significantly, *i.e.*, the coverage of PANI-ES is assumed to be small enough within the timescale of vesicle observation (~100 sec), and/or the initiation reaction and propagation reaction do not interfere each other (film-on-film growth mechanism)[31]. Growth of vesicle membrane coupled with information polymer, reaction (M5), **(Supplementary Fig. 3-1f**): Incorporation of free membrane molecules (A_{m}) in the external solution into the outer layer of the vesicle membrane ($A_{\mathrm{v}}^{\mathrm{o}}$) through PANI-ES ($P_n$) is described by a simple model reaction involving intermediate complex states between amphiphile and PANI-ES. We suppose that A_{m} is bound to PANI-ES (P_n) on the vesicle surface as A(a) **(**($A_{\mathrm{m}}P_n$) in **Supplementary Fig. 1b**) and A'(a) **(**($P_nA_{\mathrm{v}}^{\mathrm{o}}$) in **Supplementary Fig. 1b**). In this model reaction, A_{m} is transferred from the external solution into the outer layer of vesicle membrane through P_n, while $A_{\mathrm{v}}^{\mathrm{o}}$ is not transferred to the external solution through P_n. The mutual catalytic mechanism between PANI-ES (P_n) synthesis and uptake of free membrane molecules (A_{m}) describes the exponential membrane growth coupled with our artificial metabolic pathways.

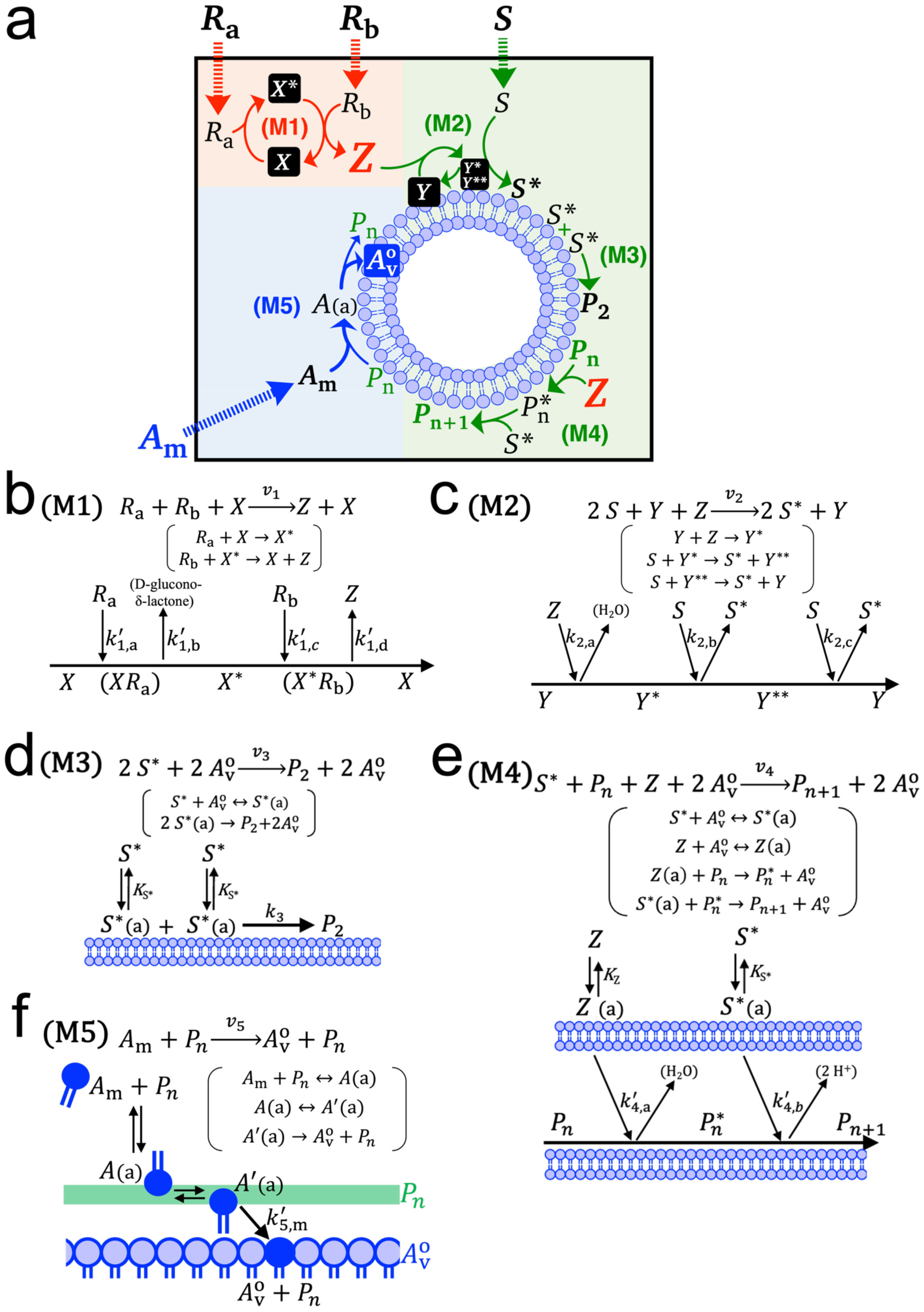
a
Ra Rb S
X* Rb (M1) Ra X Z
(M2) S Y* Y** Y S*
S* + S* (M3) P2
Pn Av° (M5) A(a) Am Pn
Pn Z P*n Pn+1 S* (M4)
Am
b (M1) Ra + Rb + X →v1 Z + X
Ra + X → X*
Rb + X* → X + Z
Ra (D-glucono-δ-lactone) Rb Z
k'1,a k'1,b k'1,c k'1,d
X (XRa) X* (X*Rb) X
c (M2) 2 S + Y + Z →v2 2 S* + Y
Y + Z → Y*
S + Y* → S* + Y**
S + Y** → S* + Y
Z (H2O) S S* S S*
k2,a k2,b k2,c
Y Y* Y** Y
d (M3) 2 S* + 2 Av° →v3 P2 + 2 Av°
S* + Av° ↔ S*(a)
2 S*(a) → P2+2Av°
S* S* KS* KS*
S*(a) + S*(a) k3 P2
e (M4) S* + Pn + Z + 2 Av° →v4 Pn+1 + 2 Av°
S* + Av° ↔ S*(a)
Z + Av° ↔ Z(a)
Z(a) + Pn → P*n + Av°
S*(a) + P*n → Pn+1 + Av°
Z S* KZ KS*
Z (a) S*(a)
(H2O) (2 H+)
k'4,a k'4,b
Pn P*n Pn+1
f (M5) Am + Pn →v5 Av° + Pn
Am + Pn
Am + Pn ↔ A(a)
A(a) ↔ A'(a)
A'(a) → Av° + Pn
A(a) A'(a) Pn
k'5,m
Av°
Av° + Pn

Supplementary Figure 3-1. Reduced model reactions of the artificial metabolic pathways.

a, Chemical scheme of the reduced model reactions (M1) – (M5) based on the actual chemical reaction scheme of the artificial metabolic pathways, (R1) – (R9), in **Fig. 1b** and **Supplementary Schemes 1**, **2**, and **Supplementary Fig. 1**. The five model reactions are assigned to three essential units; energy production (orange), M1, synthesis of information polymer (green), M2 – M4, and membrane growth (blue), M5. Thick red or green dashed arrows towards the inside of a box represent that reactants (ingredients) are supplied to the vesicles. Membrane molecules are also supplied to the vesicles (thick blue dashed arrow) and then incorporated into the membrane through PANI-ES (blue arrows). Each model reaction represents the essential process of the artificial metabolic pathways described in the text. Each symbol represents the following molecules: R_{a} (D-glucose), R_{b} (dissolved oxygen), S (aniline), S^{*} (aniline radical cation), P_2 (aniline dimer), P_n (PANI-ES chain with a degree of polymerisation $n \geq 2$), P_n^{*} (oxidised PANI-ES chain), A_{m} (free AOT molecule), $A(\mathrm{a})$ and $A'(\mathrm{a})$ (intermediate complex of AOT and PANI-ES), $A_{\mathrm{v}}^{\mathrm{o}}$ (AOT molecule located in the outer leaflet of vesicle membrane), X (enzyme GOD), Y (enzyme HRPC), and Z (energy currency H_2O_2).

b, Schematic of model reaction (M1) following the two-substrate ping-pong mechanism. v_1 is the reaction rate of M1. For **b** – **f**, the detailed explanations are described in the text.

c, Schematic of model reaction (M2) following the irreversible ping-pong mechanism. v_2 is the reaction rate of M2 concerning the consumption of Z.

d, Schematic of model reaction (M3) following the Langmuir-Hinshelwood mechanism. v_3 is the reaction rate of M3 concerning the formation of P_2.

e, Schematic of model reaction (M4) following the irreversible ping-pong mechanism combined with the Langmuir-Hinshelwood mechanism. v_4 is the reaction rate of M4 concerning the chain propagation.

f, Schematic of model reaction (M5) based on the molecular mechanism described in **Supplementary Note 1-3.** v_5 is the reaction rate of M5 concerning incorporation of membrane molecule.

3-2. Rate equations of reduced model reactions.

Rate equations, Eq.(11) – (15) in the main text, are derived based on the reduced model reactions of the artificial metabolic pathway, (M1) – (M5) (**Supplementary Fig. 3-1**), as follows:

$$v_1(t)[(\text{mol L}^{-1})\ \text{s}^{-1}] = \frac{[X]_0}{\frac{1}{k_{1,\text{a}}} + \frac{1}{k_{1,\text{b}}[R_\text{a}(t)]} + \frac{1}{k_{1,\text{c}}[R_\text{b}]}} \qquad (V1)$$

$$v_2(t)\ [(\text{mol m}^{-2})\ \text{s}^{-1}] = \frac{[Y]_0}{\frac{1}{k_{2,\text{a}}[Z(t)]} + \frac{k_{2,\text{b}} + k_{2,\text{c}}}{k_{2,\text{b}}k_{2,\text{c}}[S(t)]}} \qquad (V2)$$

$$v_3(t)\ [(\text{mol m}^{-2})\ \text{s}^{-1}] = k_3 n_\text{v} \left(\frac{K_{S^*}[S^*(t)]}{1 + K_{S^*}[S^*(t)] + K_Z[Z(t)]} \right)^2 \qquad (V3)$$

$$v_4'(t)[(\text{mol m}^{-2})\ \text{s}^{-1}] = \frac{[P(t)]}{[A(t)]} \frac{1}{\frac{1}{k'_{4,\text{a}} n_\text{v} \left(\frac{K_Z[Z(t)]}{1 + K_Z[Z(t)] + K_{S^*}[S^*(t)]} \right)} + \frac{1}{k'_{4,\text{b}} n_\text{v} \left(\frac{K_{S^*}[S^*(t)]}{1 + K_Z[Z(t)] + K_{S^*}[S^*(t)]} \right)}} \qquad (V4')$$

$$v_5(t)[(\text{mol m}^{-2})\ \text{s}^{-1}] = k'_{5,\text{m}} \frac{K'_\text{m}[A_\text{m}]}{1 + K'_\text{m}[A_\text{m}]} \frac{[E(t)]}{[A(t)]} = k_5[A_\text{m}] \frac{[E(t)]}{[A(t)]}. \qquad (V5)$$

Here the variables are the concentrations of aniline ($[S(t)]$), aniline radical cation ($[S^*(t)]$), D-glucose ($[R_\text{a}(t)]$), energy currency H_2O_2 ($[Z(t)]$), the total number of PANI-ES chains ($[P(t)]$) ($P = \sum_{n=1}^{\infty} N_n$, N_n:number of PANI-ES chains with the degree of polymerisation of $n \geq 2$), the total number of PANI-ES segments ($[E(t)]$) ($E = \sum_{n=1}^{\infty} nN_n$) that have the unit of mol L^{-1}, and the vesicle surface area exposed to the external solution per unit volume ($[A(t)]$) that have the unit of $\text{m}^2\ \text{L}^{-1}$. $[X]_0$ and $[Y]_0$ represent the total concentration of enzyme GOD (X), $[X]_0 = [X] + [X^*] + [XR_\text{a}] + [X^*R_\text{b}]$, and enzyme HRPC ($Y$), $[Y]_0 = [Y] + [Y^*] + [Y^{**}]$, respectively.

The rate equation Supplementary Eq.(V1), production of energy currency molecules, is expressed by the two-substrate ping-pong mechanism[27,28] of reaction (M1) (**Supplementary Fig. 3-1b**), where we applied steady-state approximation to $[X^*]$, $[XR_\text{a}]$, and $[X^*R_\text{b}]$. The concentration of dissolved O_2 in the reaction mixture $[R_\text{b}]$ assumes to be in equilibrium. Here the rate constants used in Supplementary Eq.(V1) are represented as follows: $k_{1,\text{a}} = \frac{k'_{1,\text{b}}k'_{1,\text{d}}}{k'_{1,\text{b}}+k'_{1,\text{d}}}$, $k_{1,\text{b}} = \frac{k'_{1,\text{b}}}{K_{1,\text{A}}}$, $k_{1,\text{c}} = \frac{k'_{1,\text{d}}}{K_{1,\text{B}}}$, where the equilibrium constants are given by $K_{1,\text{A}} = \frac{[R_\text{a}][X]}{[XR_\text{a}]}$, and $K_{1,\text{B}} = \frac{[R_\text{b}][X^*]}{[X^*R_\text{b}]}$, and the rate constants in the ping-pong mechanisms, $k'_{1,\text{a}}$, $k'_{1,\text{b}}$, $k'_{1,\text{c}}$, and $k'_{1,\text{d}}$ are shown in **Supplementary Fig. 3-1b**. The values of rate constants are obtained in the form of $k_{1,\text{a}}, k_{1,\text{b}}$, and $k_{1,\text{c}}$ from the literature[32].

The rate equation Supplementary Eq.(V2), production of aniline radical cation for the

template polymerisation, is expressed by the irreversible ping-pong mechanism[30] of reaction (M2) (**Supplementary Fig. 3-1c**), where we apply steady-state approximation to $[Y], [Y^*],$ and $[Y^{**}]$. The values of rate constants, $k_{2,\mathrm{a}}, k_{2,\mathrm{b}}$, and $k_{2,\mathrm{c}}$, are given by literatures[9,30,33–35].

The rate equation Supplementary Eq.(V3), initiation of the template polymerisation, is expressed by the Langmuir-Hinshelwood mechanism of reaction (M3) (**Supplementary Fig. 3-1d**), where two S^* molecules are firstly adsorbed on AOT vesicle membrane[5–8], and then two adsorbed molecules react to form a dimer, P_2. Here, n_v is the molar concentration of AOT molecules in the outer layer of vesicle membrane [mol m^{-2}], K_{S^*} is the adsorption equilibrium constant of aniline radical cation on the AOT membrane, which is estimated by the MD simulations for adsorption equilibrium of anilinium cation[7]. K_Z is the adsorption equilibrium constant of H_2O_2 on AOT membrane, which is assumed to have the same value as K_{S^*}. It should be noted that this value does not have a significant influence on reproducing the experimental results. The value of the rate constant k_3 = 1.0 ·10^4 s^{-1} is determined by fitting the time evolution of the PANI-ES synthesis triggered by the direct H_2O_2 addition, control experiment-0 (**Supplementary Note 2-1** and **Supplementary Fig.2-1c**), using model-4 described in **Supplementary Note 3-3(iv)**.

The rate equation Supplementary Eq.(V4'), propagation of the template polymerisation, is expressed by the irreversible ping-pong mechanism of model reaction (M4) (**Supplementary Fig. 3-1e**). For simplicity, by introducing a new rate constant k_4, we approximated the rate equation Supplementary Eq.(V4) as

$$v_4(t)[(\mathrm{mol\ m^{-2}})\ \mathrm{s^{-1}}] = k_4 n_\mathrm{v} \frac{[P(t)]}{[A(t)]} \frac{K_Z[Z(t)] \cdot K_{S^*}[S^*(t)]}{(1 + K_Z[Z(t)] + K_{S^*}[S^*(t)])(K_Z[Z(t)] + K_{S^*}[S^*(t)])} \quad (V4)$$

This rate equation, Supplementary Eq.(V4), is adopted in the text as Eq.(14). The value of the rate constant k_4 = 3.2 ·10^7 [mol^{-1} m^2 s^{-1}] is determined by fitting the time evolution of PANI-ES synthesis triggered by the direct addition of H_2O_2, control experiment-0 (**Supplementary Note 2-1** and **Supplementary Fig. 2-1c**), using model-4 described in **Supplementary Note 3-3(iv)**.

The rate equation Supplementary Eq.(V5), incorporation of membrane molecules, is obtained from the model reaction (M5) by assuming the steady-state approximation, as described in **Supplementary Note 3-1**. K'_m is the equilibrium constant for $A_\mathrm{m} + P_n \rightleftarrows A(\mathrm{a}) \rightleftarrows A'(\mathrm{a})$. It should be noted that the concentration of externally supplied AOT molecules ([A_m] = 20 mM) is constant in this work. The value of the redefined rate constant k_5 = 0.30 [mol^{-1} L s^{-1}] is determined by fitting the AOT membrane growth profile coupled with PANI-ES synthesis triggered by the direct addition of H_2O_2, control experiment-1 (**Supplementary Note 2-2** and **Supplementary Fig. 2-2c**), using model-3 described in **Supplementary Note 3-3(iii)**. The parameters appeared in the rate equations, Supplementary Eq.(V1) – (V5), with their initial values are summarised in **Supplementary Table 1** in **Supplementary Note 3-4**.

3-3. Simultaneous differential equations describing the kinetics of artificial metabolic pathways.

Based on the above discussion (**Supplementary Note 3-1** and **3-2**), the simultaneous differential equations describing the kinetics of our artificial metabolic pathways (**Fig. 1b**) are derived as model-1 in **Supplementary Note 3-3(i)**. The vesicle membrane growth (**Fig. 3b**) is described with the full model of our artificial metabolic pathways (model-1), while the time evolution of enzymatic cascade synthesis of PANI-ES (**Fig. 2e**) is described with model-2 in **Supplementary Note 3-3(ii)**, where the cascade reaction was carried out in a reaction tube with consumption of initial ingredients (aniline and D-glucose) and no external supply of AOT molecules (no membrane growth). The calculated time evolution of PANI-ES synthesis and the growth profile of AOT vesicle membrane are compared with the experimental results as shown in **Fig. 2e** and **Fig. 3b**, respectively. Preceding the calculations, the rate equations, Supplementary Eq.(V3), (V4), and (V5), need to determine the values of rate constants, k_3, k_4, and k_5 (**Supplementary Note 3-2**). Here, the rate constants k_3 and k_4 were determined by fitting the time evolution of PANI-ES synthesis obtained from control experiment-0 (**Supplementary Fig. 2-1c**) using model-3 (**Supplementary Note 3-3(iii)**). Then, the rate constant k_5 was determined by fitting the growth profile of AOT GUV membrane obtained from control experiment-1 (**Supplementary Fig. 2-2c**) using model-4 (**Supplementary Note 3-3(iv)**).

(i) Kinetic model for full artificial metabolic pathways: *Model-1*

The reduced chemical model of the full artificial metabolic pathways (**Fig. 1b**) is schematically shown in **Supplementary Fig. 3-2a** as model-1, which is represented by model reactions, (M1) – (M5) (**Supplementary Note 3-1)**. Simultaneous differential equations based on the model-1 are given by

$$\frac{\mathrm{d}[P(t)]}{\mathrm{d}t} = v_3(t)\,[A(t)] \tag{Ia}$$

$$\frac{\mathrm{d}[E(t)]}{\mathrm{d}t} = \big(2\,v_3(t) + v_4(t)\big)\,[A(t)] \tag{Ib}$$

$$\frac{\mathrm{d}[S^*(t)]}{\mathrm{d}t} = \big(2\,v_2(t) - 2\,v_3(t) - v_4(t)\big)\,[A(t)] \tag{Ic}$$

$$\frac{\mathrm{d}[Z(t)]}{\mathrm{d}t} = v_1(t) - \big(v_3(t) + v_4(t)\big)\,[A(t)] \tag{Id}$$

$$\frac{\mathrm{d}[A_\mathrm{v}(t)]}{\mathrm{d}t} = v_5(t)\,[A(t)] \tag{Ie}$$

$$[A(t)] = \frac{1}{2}[A_\mathrm{v}(t)]\,N_\mathrm{A}\,a_\mathrm{v} \tag{If}$$

$$\frac{\mathrm{d}[S(t)]}{\mathrm{d}t} = \frac{\mathrm{d}[R_\mathrm{a}(t)]}{\mathrm{d}t} = 0, \tag{Ig}$$

which describes the membrane growth of AOT GUVs coupled with the synthesis of PANI-ES with the optimised cascade reaction condition, *i.e.*, artificial metabolic pathway. The experimentally observed membrane growth (red circles in **Fig. 3b**) is well reproduced by $[A(t)]/[A(0)]$ with model-1 (red line in **Fig. 3b**) without free parameters. It should be noted that the rate constants, k_3, k_4, and k_5, were pre-determined by the control experiments-0 and -1 (**Supplementary Note 2, 3-3(iii)** and **(iv)**). The important notice is that the three essential units (energy production, synthesis of information polymer, and membrane growth) are unified into the full artificial metabolic pathways, model-1.

(ii) Kinetic model for PANI-ES synthesis in cascade reaction system: *Model-2*

The time dependence of PANI-ES synthesis in the cascade reaction system **(Fig. 2e)** was measured without the external supply of D-glucose, aniline, and AOT molecules, *i.e.*, no membrane growth. The reduced chemical pathway for this PANI-ES synthesis scheme (model-2), (M1) – (M4), is schematically shown in **Supplementary Fig. 3-2b**. Simultaneous differential equations based on the chemical pathway, model-2, are expressed by

$$\frac{\mathrm{d}[P(t)]}{\mathrm{d}t} = v_3(t)\,[A(t)] \tag{IIa}$$

$$\frac{\mathrm{d}[E(t)]}{\mathrm{d}t} = \left(2\,v_3(t) + v_4(t)\right)[A(t)] \tag{IIb}$$

$$\frac{\mathrm{d}[S^*(t)]}{\mathrm{d}t} = \left(2\,v_2(t) - 2\,v_3(t) - v_4(t)\right)[A(t)] \tag{IIc}$$

$$\frac{\mathrm{d}[Z(t)]}{\mathrm{d}t} = v_1(t) - \left(v_3(t) + v_4(t)\right)[A(t)] \tag{IId}$$

$$\frac{\mathrm{d}[A_\mathrm{v}(t)]}{\mathrm{d}t} = 0 \tag{IIe}$$

$$[A(t)] = \frac{1}{2}[A_\mathrm{v}(t)]\,N_\mathrm{A}\,a_\mathrm{v} \tag{IIf}$$

$$\frac{\mathrm{d}[S(t)]}{\mathrm{d}t} = -2\,v_2(t)\,[A(t)] \tag{IIg}$$

$$\frac{\mathrm{d}[R_\mathrm{a}(t)]}{\mathrm{d}t} = -v_1(t)\,. \tag{IIh}$$

Since AOT molecules (A_m) are not externally supplied to the system, AOT vesicles show no growth (Supplementary Eq.(IIe)). D-glucose and aniline in the initial external solution decrease as the reaction progresses (Supplementary Eq.(IIg) and (IIh)) since D-glucose (R_a) and aniline

(S) are not externally supplied. The time evolution of PANI-ES synthesis simulated by $[E(t)]/[E(24\mathrm{h})]$ with model-2 well reproduces the experimental result without free parameters as shown in **Fig. 2e**. The parameters k_3 and k_4 are pre-determined by the control experiment-0 (**Supplementary Note 2** and **3-3(iii)**).

(iii) Kinetic model for PANI-ES synthesis without "energy production" unit: *Model-3*

To examine the roles of the energy production unit in the artificial metabolic pathways, we measured the time evolution of PANI-ES synthesis without the energy production unit (M2) – (M4), control experiment-0, as shown in **Supplementary Note 2-1** and **Supplementary Fig. 2-1c.** The reduced chemical pathway for the control experiment-0 (model-3) is schematically shown in **Supplementary Fig. 3-2c**. The simultaneous differential equations describing this chemical scheme, model-3, are given by

$$\frac{\mathrm{d}[\mathrm{P}(t)]}{\mathrm{d}t} = v_3(t)\,[A(t)] \tag{IIIa}$$

$$\frac{\mathrm{d}[E(t)]}{\mathrm{d}t} = \big(2\,v_3(t) + v_4(t)\big)\,[A(t)] \tag{IIIb}$$

$$\frac{\mathrm{d}[S^*(t)]}{\mathrm{d}t} = \big(2\,v_2(t) - 2\,v_3(t) - v_4(t)\big)\,[A(t)] \tag{IIIc}$$

$$\frac{\mathrm{d}[Z(t)]}{\mathrm{d}t} = -\big(v_3(t) + v_4(t)\big)\,[A(t)] \tag{IIId}$$

$$\frac{\mathrm{d}[A_\mathrm{v}(t)]}{\mathrm{d}t} = 0 \tag{IIIe}$$

$$[A(t)] = \frac{1}{2}[A_\mathrm{v}(t)]\,N_\mathrm{A}\,a_\mathrm{v} \tag{IIIf}$$

$$\frac{\mathrm{d}[S(t)]}{\mathrm{d}t} = -2\,v_2(t)\,[A(t)] \tag{IIIg}$$

The PANI-ES synthesis is carried out using aniline (S) and H_2O_2 (Z) that are initially present in the reaction solution. Thus, aniline and H_2O_2 decrease as the reaction progresses (Supplementary Eq.(IIIg) and (IIId)), and the total surface area of vesicles in the system is constant (Supplementary Eq.(IIIe)) due to no supply of AOT molecules (A_m). The time evolution of PANI-ES synthesis simulated by $[E(t)]/[E(24\mathrm{h})]$ with model-3 well reproduces the experimental profile as shown in **Supplementary Fig. 2-1c**, which gives the rate constants $k_3 = 1.0 \cdot 10^4$ [s^{-1}] and $k_4 = 3.2 \cdot 10^7$ [$\mathrm{mol}^{-1}\,\mathrm{m}^2\,\mathrm{s}^{-1}$].

(iv) Kinetic model for membrane growth of AOT GUV coupled with PANI-ES synthesis without "energy production" unit: *Model-4*

To examine the roles of the energy production unit in the artificial metabolic pathways, we measured the growth of AOT membrane coupled with PANI-ES synthesis without the energy production unit, control experiment-1, as shown in **Supplementary Note 2-2** and **Supplementary Fig. 2-2c**. The reduced chemical pathway for the control experiment-1 (model-4) is schematically shown in **Supplementary Fig. 3-2d**. The simultaneous differential equations describing this chemical scheme, model-4, are given by

$$\frac{\mathrm{d}[P(t)]}{\mathrm{d}t} = v_3(t)\,[A(t)] \tag{IVa}$$

$$\frac{\mathrm{d}[E(t)]}{\mathrm{d}t} = \left(2\,v_3(t) + v_4(t)\right)\,[A(t)] \tag{IVb}$$

$$\frac{\mathrm{d}[S^*(t)]}{\mathrm{d}t} = \left(2\,v_2(t) - 2\,v_3(t) - v_4(t)\right)\,[A(t)] \tag{IVc}$$

$$\frac{\mathrm{d}[Z(t)]}{\mathrm{d}t} = \frac{\mathrm{d}[S(t)]}{\mathrm{d}t} = 0 \tag{IVd}$$

$$\frac{\mathrm{d}[A_\mathrm{v}(t)]}{\mathrm{d}t} = v_5(t)\,[A(t)] \tag{IVe}$$

$$[A(t)] = \frac{1}{2}[A_\mathrm{v}(t)]\,N_\mathrm{A}\,a_\mathrm{v} \tag{IVf}$$

The energy currency molecule H_2O_2 (Z) is directly supplied instead of producing it in the energy production pathway (see **Supplementary Fig. 3-2d**). The time evolution of the membrane surface area simulated by $[A(t)]/[A(0)]$ with model-4 well reproduces the experimental profile as shown in **Supplementary Fig. 2-2c**, which gives the rate constant $k_5 = 0.30$ [$\mathrm{mol^{-1}\,L\,s^{-1}}$].

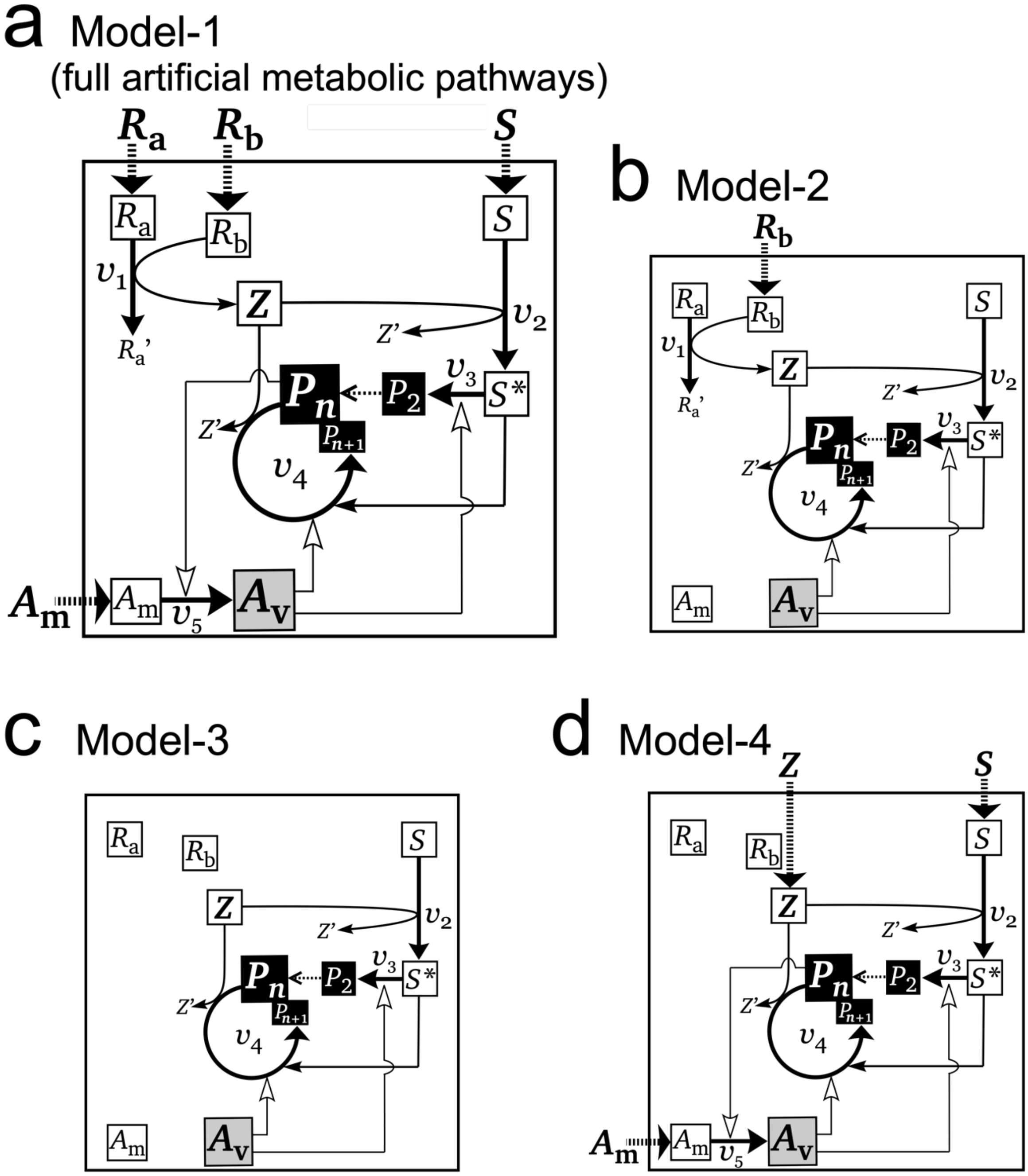

Supplementary Figure 3-2. Schematics of chemical pathways for models 1–4. Chemical pathways in each model are schematically shown by using the following symbols: Solid arrows represent chemical transformations of substances by reactions, a solid circular arrow at "P_n" represents template polymerisation of PANI-ES assisted by the vesicle membrane, open arrows indicate which molecule assists which reactions, and thick dashed arrows represent the continuous supply of ingredients to the chemical system. "v_i" (i = 1–5) beside the solid arrows represent the reaction rate of each chemical pathway. Each character in a box represents an essential molecule of artificial metabolic

pathways as follows: aniline (S), aniline radical cation (S^*), D-glucose (R_a), gluconic acid (R'_a), dissolved oxygen (R_b), energy currency H_2O_2 (Z), H_2O (Z'), aniline dimer PADPA (P_2), PANI-ES chain with a degree of polymerisation n (P_n), AOT molecules forming vesicle membrane (A_v), and free AOT molecules in the external solution (A_m).

a, Reduced chemical pathway for the full artificial metabolic pathways of our synthetic minimal cell, (M1)–(M5), **Supplementary Fig.3-1a**: Model-1. Model-1 is described by the differential equations Supplementary Eq.(Ia) – (Ig) in **Supplementary Note 3-3(i)**, which reproduces the experimental result on AOT membrane growth under micro-injections shown in **Fig. 3b**.

b, Reduced chemical pathway for PANI-ES synthesis in the cascade reaction system without membrane growth, (M1) – (M4): Model-2. In this model, no ingredients except for R_b are externally supplied during the reaction, and the chemical system is driven by initially added R_a and S in the solution. Model-2 is described by the differential equations Supplementary Eq.(IIa)–(IIh) in **Supplementary Note 3-3(ii)**, which reproduces the experimental result on the time-evolution of enzymatic cascade PANI-ES synthesis in a micro-tube as shown in **Fig. 2e**.

c, Reduced chemical pathway for PANI-ES synthesis without "energy production" and "membrane growth", (M2) – (M4): Model-3. In this model, no ingredients are externally supplied during the reaction, and the chemical system is driven by initially added Z and S (control experiment-0). The model-3 is described by the differential equations Supplementary Eq.(IIIa)–(IIIg) in **Supplementary Note 3-3(iii)**, which are used to determine the values of rate constants k_3 and k_4 by fitting the experimental results on the time-evolution of HRPC/H_2O_2-catalysed PANI-ES synthesis shown in **Supplementary Fig. 2-1c**.

d, Reduced chemical pathway for membrane growth coupled with PANI-ES synthesis without "energy production" unit, (M2) – (M5): Model-4. The chemical system is driven by the continuous supply of Z and S (control experiment-1). Model-4 is described by the differential equations Supplementary Eq.(IVa)–(IVf) in **Supplementary Note 3-3(iv)**, which are used to determine the value of rate constant k_5 by fitting the membrane growth coupled with the HRPC/H_2O_2-catalysed PANI-ES synthesis shown in **Supplementary Fig. 2-2c**.

3-4. Parameter list of the kinetic model.

Supplementary Table 1. The parameters and their values applied to the kinetic model.

	Parameter	Value	Comment	Reference
X (GOD)	$[X]_0$	1.0 ·10^{-6} [M]	Initial concentration of GOD.	From experimental conditions.
	$k_{1,a}$	1.0 ·10^{3} [s^{-1}]	Obtained at pH = 4.0, in potassium citrate buffer. $k_{1,a} = \frac{k'_{1,b}k'_{1,d}}{k'_{1,b}+k'_{1,d}}$ (see **Supplementary Note 3-2**)	32
	$k_{1,b}$	1.5 ·10^{4} [M^{-1} s^{-1}]	Obtained at pH = 4.0, in potassium citrate buffer. $k_{1,b} = \frac{k'_{1,b}}{K_{1,A}}$ (see **Supplementary Note 3-2**)	32
	$k_{1,c}$	1.9 ·10^{6} [M^{-1} s^{-1}]	Obtained at pH = 4.0, in potassium citrate buffer. $k_{1,c} = \frac{k_{1,d}}{K_{1,B}}$ (see **Supplementary Note 3-2**)	32
	$[R_a]_0$	0.100 [M]	Initial concentration of D-glucose for the optimised cascade reaction.	From experimental condition.
		0 [M]	Initial concentration of D-glucose for HRPC-catalysed reaction system with direct H_2O_2 addition.	
	$[R_b]_0$	0.23·10^{-3} [M] (saturated value)	O_2 is consumed for H_2O_2 production and supplied externally during the reaction. The concentration of O_2 in the reaction mixture assumes to be in equilibrium at 10% (in rotary mixed microtube system) or at 3% (in micro-injection system) of the saturated value.	36,37
Y (HRPC)	$[Y]_0$	3.04 ·10^{-9} [mol m^{-2}] (0.92 ·10^{-6}	Initial concentration of HRPC. Concentration of HRPC in reaction solution (0.92·10^{-6} [M]) is converted to	From experimental condition.

		[M])	concentration on the surface of vesicle membrane [mol m^{-2}] by using the value of $[A]_0$, see below.	
	$k_{2,\mathrm{a}}$	1.7 ·10^{7} [M^{-1} s^{-1}]	This value is obtained at pH = 7.0 in various solutions.	9,30,33
	$k_{2,\mathrm{b}}$	2.4 ·10^{5} [M^{-1} s^{-1}]	This value is obtained at pH = 7.0 in phosphate buffer.	30,34
	$k_{2,\mathrm{c}}$	8.6 ·10^{4} [M^{-1} s^{-1}]	This value is obtained at pH = 7.0 in sodium phosphate buffer.	30,35
	HRPC activity	~ 20%	At pH = 4.3, HRPC activity decreases to ~20% of the activity at pH = 7.	38
AOT	N_A	6.02 ·10^{23} [mol^{-1}]	The Avogadro constant.	
	a_v	0.67·10^{-18} [m^{2}]	Surface area per single AOT molecule in vesicle membrane.	39
	n_v	2.48 ·10^{-6} [mol m^{-2}]	Density of AOT molecules on the surface of vesicle membrane given by a_v^{-1}.	39
	cvc	1.5·10^{-3} [M]	Concentration of AOT molecules present as the single molecule state, *i.e.*, critical vesiculation concentration, in 20 mM NaH_2PO_4 solution (pH = 4.3).	20
	$[A_\mathrm{v}]_0$	1.5·10^{-3} [M]	Concentration of AOT molecules forming vesicles in 20 mM NaH_2PO_4 solution (pH = 4.3). (AOT total concentration in vesicle suspension [mM] – cvc [mM])	20
	$[A]_0$	3.03 ·10^{2} [m^{2} L^{-1}]	Vesicle surface area per 10^{-3} m^{3} (1 L) dispersed in the reaction mixture. Given by: $[A_\mathrm{v}]_0$[M] · 1/2 · N_A[mol^{-1}] · a_v[m^{2}].	From experimental condition.
	$[A_\mathrm{m}]$	20·10^{-3} [M]	Concentration of free AOT molecules supplied to the vesicles with micro-injection.	
	k_5	0.30 [M^{-1} s^{-1}]	Rate constant for the incorporation of AOT molecules, which was determined	see **Supp. Note 2-2.**

			by control experiment-1 using model-4.	
S (Aniline)	$[S]_0$	$4.0 \cdot 10^{-3}$ [M]	Initial concentration of aniline.	From experimental condition.
	$[S^*]_0$	0 [M]	Initial concentration of aniline radical cation.	
	K_{S^*}	2.3 [M^{-1}]	Equilibrium constant of the Langmuir adsorption of aniline radical cation. The value is assumed to be the same with that of anilinium cation and estimated from the outcome of MD simulation for anilinium cation adsorbed on AOT membrane.	7
Z (H_2O_2)	$[Z]_0$	0 [M]	Initial concentration of H_2O_2 for the optimised cascade reaction system.	From experimental condition.
		$4.5 \cdot 10^{-3}$ [M]	Initial concentration of H_2O_2 for the HRPC-catalysed reaction system coupled with direct addition of H_2O_2.	
	K_Z	2.3 [M^{-1}]	Equilibrium constant of Langmuir adsorption of H_2O_2. The value is assumed to be the same with K_{S^*}.	
PANI-ES	k_3	$1.0 \cdot 10^{4}$ [s^{-1}]	Rate constant on initiation reaction of PANI-ES, which was determined by control experiment-0 using model-3.	see **Supp. Note 2-1** and **3-3(iii).**
	k_4	$3.2 \cdot 10^{7}$ [mol^{-1} m^2 s^{-1}]	Rate constant on elongation reaction of PANI-ES, which was determined by control experiment-0 using model-3.	see **Supp. Note 2-1** and **3-3(iii).**
	$[P]_0$	0 [M]	Initial concentration of PANI-ES chain.	
	$[E]_0$	0 [M]	Initial concentration of PANI-ES segments.	

Supplementary Note 4.

Time dependence of ingredient (D-glucose and aniline) concentrations during the progress of artificial metabolic reactions: experiment vs kinetic model.

Here we compare the time dependence of ingredients (D-glucose and aniline) concentrations obtained by the experiment with the theoretical predictions. The chemical pathway is the same as the enzymatic cascade synthesis of PANI-ES, model-2, (M1) – (M4) (**Fig. 2e**), where the polymerisation reaction is triggered by D-glucose, dissolved oxygen, and aniline initially present in a closed micro-tube.

First, we quantified experimentally the concentration of D-glucose and aniline during the reaction. The quantification procedures are described in our previous study[15]. The concentration of aniline (black squares in **Supplementary Fig. 4a**) was determined spectrophotometrically by withdrawing small volumes from the reaction mixtures and then adding them to acetonitrile. After centrifugation, the characteristic absorption intensity at λ = 238 nm of the supernatant solution was recorded and then compared with the calibration curve obtained with known amounts of aniline. The concentration of D-glucose in the enzymatic cascade reaction mixture (black squares in **Supplementary Fig. 4b**) was determined by separating GOD and HRPC from the reaction mixture with ultrafiltration and then using the GOD/HRPC/$ABTS^{2-}$ assay[15]. The purified enzyme-free reaction mixture containing D-glucose was added to another solution containing GOD, HRPC, and 2,2′-azino-bis(3-ethylbenzothiazoline-6-sulfonate), abbreviated as $ABTS^{2-}$. Then, the enzymatically produced H_2O_2 from the remaining D-glucose oxidises $ABTS^{2-}$, which shows a linear increase in absorption intensity at λ = 414 nm during the first 180 sec. This absorption intensity slope against time was taken as a measure for the amount of D-glucose, compared with the calibration curve obtained with known amounts of D-glucose.

Second, the time-dependence of aniline ($S(t)$, green line in **Supplementary Fig. 4a**) and D-glucose ($R_\mathrm{a}(t)$, orange line in **Supplementary Fig. 4b**) concentrations was calculated by numerically solving the differential equations Supplementary Eq.(IIa) – (IIh) based on model-2 (**Supplementary Note 3-2(ii)**). It should be noted that the rapid enzyme inactivation of GOD and HRPC during the cascade reaction[15] was considered in the calculation, assuming the decay rate as $[X]_0 \rightarrow [X]_0 \cdot 2^{-t[s]/60}$ and $[Y]_0 \rightarrow [Y]_0 \cdot 2^{-t[s]/60}$. The theoretical prediction successfully describes the reactants D-glucose ($R_\mathrm{a}(t)$) and aniline ($S(t)$) quantitatively during the reaction. The agreement between the kinetic model and the experimental results on time evolutions of ingredients (**Supplementary Fig. 4a** and **b**), synthesis of information polymer (**Fig. 2e**), and vesicle membrane growth (**Fig. 3b**) supports that the unity of three units of the artificial metabolic pathways was successful and well-described by the reduced model reactions (M1) – (M5).

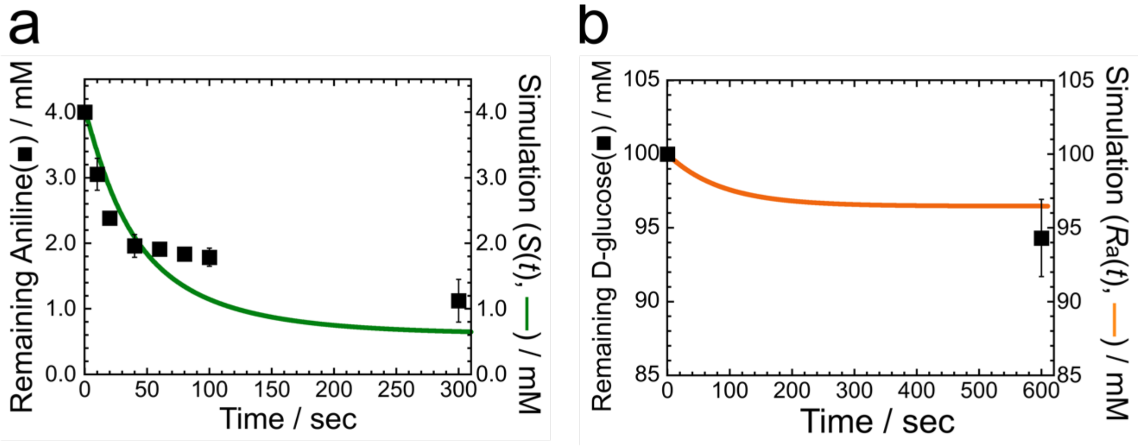

Supplementary Figure 4. Time dependence of ingredient (D-glucose and aniline) concentrations during the progress of artificial metabolic reactions.

Time-dependent changes in D-glucose (R_a) and aniline (S) concentrations were measured in the same experimental condition as the time-evolution of enzymatic cascade synthesis of PANI-ES (**Fig. 2e**). The experimental results were compared with the theoretical predictions by using the reduced chemical pathway for PANI-ES synthesis, (M1) – (M4); model-2, described by the differential equations Supplementary Eq.(IIa)–(IIh) in **Supplementary Note 3-3(ii)**. The error bars represent standard deviations obtained from two or three measurements. It should be noted that the horizontal time scales are different between each figure.

a, Time-dependence of experimental aniline concentration (black squares) compared with the theoretical prediction (green line).

b, Time-dependence of experimental D-glucose concentration (black squares) compared with the theoretical prediction (orange line).

Supplementary Note 5.

Osmotic inflation of AOT vesicles.

A volume recover of offspring vesicles to that of their mother vesicle is an indispensable element to attain the recursive vesicle reproduction cycle. An osmotic swelling (applying an osmotic pressure difference between the inside and outside of GUV membrane) is a technique to increase the vesicle volume. However, when the total amount of osmolytes encapsulated in the GUV is constant, the osmotic pressure difference decreases with time due to the inflow of water, which leads to the cessation of osmotic swelling. Therefore, for the sustainable volume increase of GUVs, it is necessary to keep the total concentration of osmolytes inside the GUVs constant against the volume increase. In addition, the external solution of GUVs needs to contain sufficient free membrane molecules to be incorporated into the membrane, otherwise, the membrane tension due to the volume increase will fracture the membrane[40]. Here we show a method for vesicle inflation in the recursive reproduction cycle of our synthetic minimal cell.

AOT GUVs encapsulating D-sucrose solution were brought into the solution containing D-fructose and AOT (**Supplementary Fig. 5a**). The AOT membrane is permeable to D-fructose but less permeable to D-sucrose. (**Supplementary Fig. 5b**). The osmotic drag couples the inflow of D-fructose with the inflow of water, causing the vesicle to swell, while AOT molecules in the external solution incorporate into the AOT membrane to release the membrane tension induced by volume inflation. Thus, the asymmetrical permeation maintains the osmotic pressure difference over time, which results in long-term inflation of AOT GUVs.

The experimental setup was as follows: AOT GUV suspension (20 mM AOT) was prepared in 20 mM NaH_2PO_4 solution containing 100 mM D-sucrose (pH = 4.3) by the gentle hydration method (see **Methods**). The 20 mM AOT GUV suspension was diluted to 3.0 mM AOT with the same NaH_2PO_4/D-sucrose solution, and then a few of AOT GUVs with a diameter of ~15 μm were trapped in a micro-pipette VacuTip II (inner diameter of 60 μm) using a CellTram Vario (Eppendorf, Germany) and then carefully transferred into the microscope sample chamber (glass-bottom dish D11130H: Matsunami, Japan) filled with 2.0 mL of 20 mM NaH_2PO_4 solution (pH = 4.3) containing 100 mM D-fructose and 3.0 mM AOT (cvc ~1.5 mM), see **Supplementary Fig. 5a**. The NaH_2PO_4 solution containing D-fructose and AOT was sonicated for 5 min at room temperature using a Branson Sonifier model 150 (Emerson, USA) and pressed through a 0.2 μm polypropylene filter Puradisc 25 PP (GE Healthcare, UK) before use.

After the transfer, the sample chamber was quickly covered by a plastic lid to prevent water flow induced by airflow. The transferred GUVs containing 100 mM D-sucrose were located at the bottom of the chamber due to the difference in the specific gravity. The inflation of GUVs was recorded at room temperature ($T \sim 25^{\circ}$C) with a phase-contrast light microscope (see **Methods**).

The location of the vesicle boundary was determined by the maximal gradient of intensity (grey level) from the image stacks, and the shape of GUVs was approximated by a sphere. From the measured radius of the GUV, the volume of a spherical vesicle was calculated. Image processing and analysis were mostly performed by using the Fiji (ImageJ) package[41].

The phase-contrast microscope image of an AOT GUV immediately after the transfer (0 sec) is compared with that after 600 sec in **Supplementary Fig. 5c**, which demonstrates the volume increase of the GUV. The time-dependent volume changes of three AOT GUVs with initial diameters of 14.6±0.1 μm are plotted as orange circles, blue triangles, and green squares in **Supplementary Fig. 5d**. The volume of AOT GUVs increased up to about 1.7 times during the observation times (~700 s). The observed volume increase of the AOT GUVs is described by

$$\frac{\mathrm{d}n_\mathrm{f}(t)}{\mathrm{d}t} = P_\mathrm{f} A(t) \left(c_\mathrm{ext} - \frac{n_\mathrm{f}(t)}{V(t)}\right) \tag{S5-1}$$

$$\frac{\mathrm{d}V(t)}{\mathrm{d}t} = v_\mathrm{w} \frac{\mathrm{d}n_\mathrm{w}(t)}{\mathrm{d}t} = P_\mathrm{w} A(t) v_\mathrm{w} \left(\frac{n_\mathrm{f}(t) + n_\mathrm{s}}{V(t)} - c_\mathrm{ext}\right), \tag{S5-2}$$

where we assume that AOT molecules in the external solution are instantly incorporated into the vesicle membrane to relax the membrane tension. Here $n_\mathrm{f}, n_\mathrm{w},$ and n_s are the amount of D-fructose (permeable), water (permeable), and D-sucrose (impermeable) molecules encapsulated in the AOT GUV, respectively. P_f and P_w are the permeability of D-fructose and water against AOT membrane, A and V are the surface area and the volume of the spherical AOT GUV, $c_\mathrm{ext}(= 0.100\ \mathrm{M})$ is the concentration of D-fructose in the external solution, and $v_\mathrm{w}(= 1.8 \cdot 10^{-2}\ \mathrm{L\ mol^{-1}})$ is the molar volume of water molecules. The observed volume growth profile is fitted by the simultaneous differential equations Supplementary Eq.(S5-1) and (S5-2) with the fitting parameters $P_\mathrm{w} = 2.3 \cdot 10^{-4}\ \mathrm{m\ s^{-1}}$ and $P_\mathrm{f} = 3.0 \cdot 10^{-9}\ \mathrm{m\ s^{-1}}$ (red solid line in **Supplementary Fig. 5d**).

For reference, the volume increase in AOT GUV induced solely by the encapsulated impermeable osmolyte is plotted as grey circles in **Supplementary Fig. 5d**. Here the AOT GUV encapsulating 100 mM sucrose (impermeable) was transferred into the 20 mM NaH_2PO_4 solution (pH = 4.3) containing 3.0 mM AOT and 90 mM sucrose (impermeable), instead of 100 mM D-fructose (permeable). This sucrose/sucrose condition, in contrast to the sucrose/fructose condition, results in a constant total amount of osmolytes encapsulated in a GUV, *i.e.*, the inflation of GUVs is not sustainable. Due to the initially imposed osmotic pressure difference, the AOT GUV showed a rapid volume increase immediately after the transfer (0 – 30 sec). However, since the total amount of osmolyte encapsulated in the GUV was kept constant, the water inflow rapidly reduced the osmotic pressure difference between inside and outside the membrane, which led to the cessation of inflation in ~80 sec. This short-term volume increase is solely described by Supplementary Eq.(S5-2), where we assume $n_\mathrm{f}(t) = 0$, $n_\mathrm{s}/V(t = 0) = 0.100\ \mathrm{M}$, and

concentration of D-sucrose in the external solution $c_{ext} = 0.090$ M (grey solid line in **Supplementary Fig. 5d**).

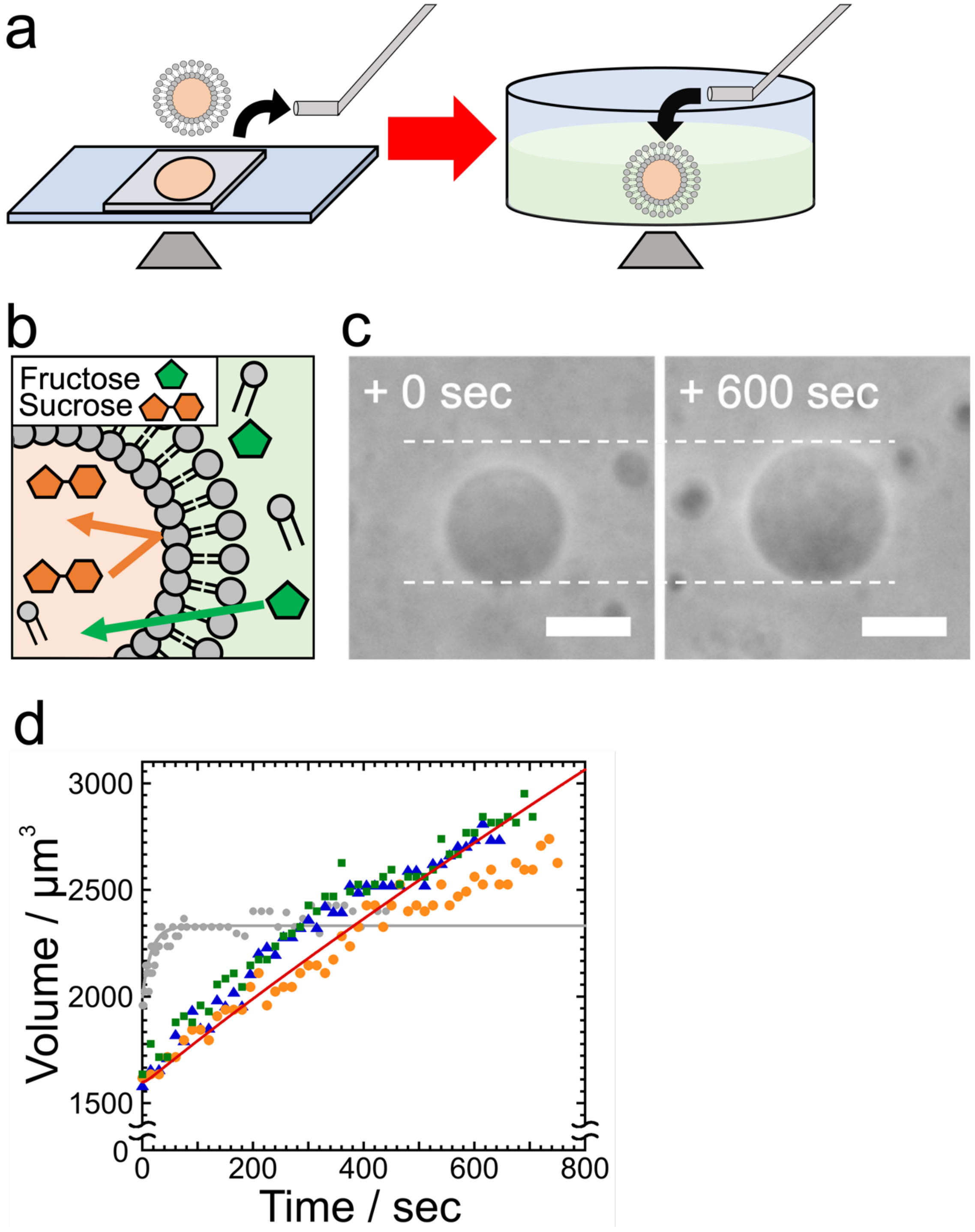

Supplementary Figure 5. Inflation of AOT GUVs using asymmetric permeation.

a, Schematic of experimental procedure for osmotic swelling of AOT GUVs. Left: AOT GUV suspension prepared in 20 mM NaH_2PO_4 solution (pH = 4.3) containing 100 mM D-sucrose and 3.0 mM AOT. A few AOT GUVs with diameter of ~15 μm were trapped in a micro-pipette. Right: The trapped AOT GUVs were carefully transferred into the sample chamber filled with 2.0 mL of 20 mM NaH_2PO_4 solution (pH = 4.3) containing 100 mM D-fructose and 3.0 mM AOT.

b, Schematic of vesicle inflation using two types of osmolytes with asymmetric membrane permeability (D-fructose and D-sucrose). The AOT membrane is permeable to D-fructose (outside) but less permeable to D-sucrose (inside). This asymmetrical permeation maintains the osmotic pressure difference between the inside and outside of the GUV, which results in long-term inflation of AOT GUVs.

c, Phase contrast microscope images of an AOT GUV swollen by two types of osmolytes with asymmetric membrane permeability (D-sucrose and D-fructose), immediately (0 sec) and 600 sec after the transfer. The elapsed time after the transfer is indicated in each image. Length of the scale bars: 10 μm.

d, Volume inflation of AOT GUV caused by impermeable D-sucrose and permeable D-fructose (orange circles, blue triangles, and green squares), where AOT GUVs encapsulating 100 mM D-sucrose was transferred into the solution containing 100 mM D-fructose and 3 mM AOT. The three plots represent the three independent observations. The initial diameters of the AOT GUVs were 14.6 ± 0.1 μm. The red line is a fitting curve obtained from the simultaneous differential equations Supplementary Eq.(S5-1) and (S5-2), yielding the permeability of water and D-fructose against AOT membrane of $P_\mathrm{w} = 2.3 \cdot 10^{-4}\ \mathrm{m\ s^{-1}}$ and $P_\mathrm{f} = 3.0 \cdot 10^{-9}\ \mathrm{m\ s^{-1}}$, respectively. For reference, volume inflation of AOT GUV caused solely by the encapsulated impermeable osmolyte (grey circles) is plotted: an AOT GUV encapsulating 100 mM D-sucrose was transferred into the solution containing 90 mM D-sucrose and 3 mM AOT. The grey line is a fitting curve obtained from the differential equation Supplementary Eq.(S5-2) with $P_\mathrm{w} = 1.0 \cdot 10^{-4}\ \mathrm{m\ s^{-1}}$.

Supplementary Note 6.

Size distribution of AOT + Chol daughter vesicles after each division.

We analysed a total of 47 total observations of binary AOT + Chol (9/1) GUV growth/division without the osmic inflation mechanism (**Fig. 4a** and **4b**) and a total of 50 observations about the recursive reproduction of binary AOT + Chol (9/1) GUVs (*i.e.*, the synthetic minimal cell system) (**Fig. 5a** and **5c**). A mother GUV produces two daughter GUVs through each growth/division cycle, and the two daughter GUVs should have the same size when the mother GUV successfully deforms to a "symmetric limiting shape" at reduced volume v = 0.7 and reduced spontaneous curvature c_0 ~ 3 (see **Discussion** section). However, we observed the size difference of two daughter GUVs in both vesicle growth/division systems, as shown in **Supplementary Fig. 6a** (for the "growth/division without the volume inflation system") and **6b** (for the "recursive reproduction system"). Here the radii of the larger GUV, R_L, (red circles) and of the smaller GUV, R_S, (blue diamonds) of the two daughter GUVs just after the division are normalised by the radius of their original mother GUV of the 1st generation, R_M, and plotted R_L/R_M and R_S/R_M for each generation. Both **Supplementary Fig. 6a** and **6b** clearly show that the vesicle divisions are generally asymmetric, where R_L/R_M has ~ 0.8 and R_S/R_M ~ 0.5 for the "growth/division without volume inflation system", whereas R_L/R_M ~ 0.9 and R_S/R_M ~ 0.5 for the "recursive reproduction system". An initially spherical binary AOT + Chol (9/1) GUV with R_0 =10 μm has v = 1.0 and c_0 ~ 0.02. The reduced volume (v) of a GUV varies with time according to the balance between the membrane growth rate and the volume inflation rate of a GUV. The reduced spontaneous curvature (c_0) also varies according to the uptake rate of the membrane molecules into the outer leaflet of the vesicle bilayer and the flip-flop rate of the membrane molecules between the inner and the outer leaflets. We sometimes observed symmetric divisions by adjusting the micro-injection flows manually, as seen in **Fig. 4a** (*#2a* and *#2b* at "+26 sec"; *#3a* and *#3b* at "+57 sec"), but it requires further elaborations in the experimental setup to systematically control v and c_0 to attain "symmetric vesicle division" every time.

From the viewpoint of the recursive nature of the vesicle reproduction, we can find a significant difference between the "growth/division without volume inflation system" and the "recursive reproduction system". In the "growth/division without volume inflation system" (**Supplementary Fig. 6a**), the size of the offspring GUVs decreased with increasing generation compared to their original mother GUV. In contrast, the larger daughter GUVs in the recursive reproduction system (red circles in **Supplementary Fig. 6b**) kept their sizes closer to that of their original mother GUV due to the volume recovery during their growth and division process. It should be noted that the radii of the larger and smaller daughter GUVs were measured just after they were produced. They recovered their mother vesicle size until the next growth and division

cycle began (see **Fig. 5b**), *i.e.*, recursive reproduction. In addition, the sizes of smaller daughter GUVs were less recovered than the larger GUVs. Since the volume increase rate determined by the osmotic inflation mechanism is proportional to the surface area of the GUVs (Eqs.(17) and (18) in **Discussion** section), the smaller GUVs take more time to recover their sizes to their initial mother GUVs.

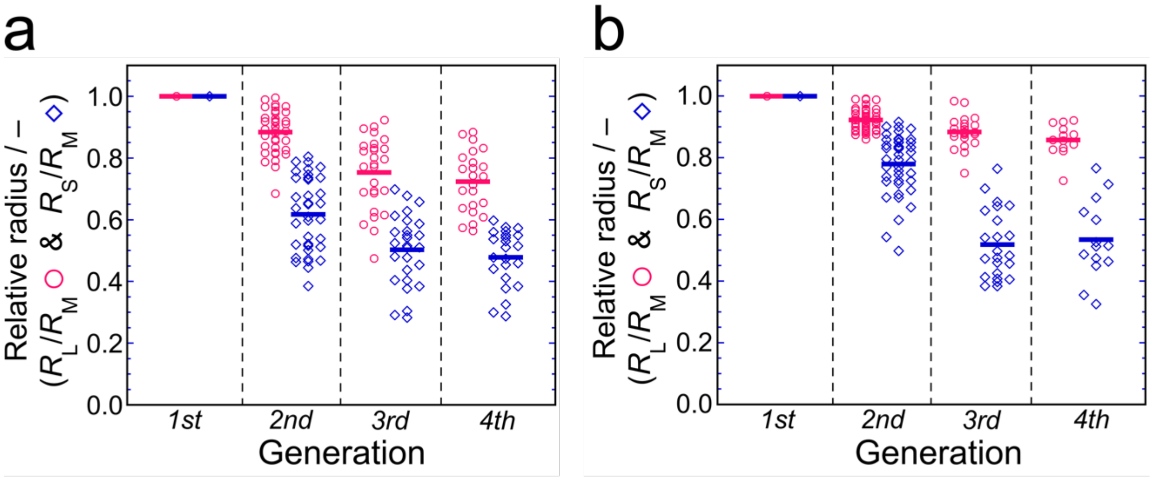

Supplementary Figure 6. Size distribution of two daughter GUVs just after the division in each generation for (a) the "growth/division without volume inflation system" and (b) the "recursive reproduction system".

The radii of the two daughter GUVs (larger GUV, R_L, and smaller GUV, R_S) after each division are normalised by the initial radius of the mother GUV, R_M, in the first generation. The relative radii, R_L/R_M (red circles) and R_S/R_M (blue diamonds), are plotted for each generation. The red and blue solid lines represent the averaged radii in each generation.

a, Relative radii of daughter GUVs just after vesicle division when AOT micelles and SDBS + Chol mixed micelles were supplied to binary AOT + Chol (9/1) GUVs under the membrane-assisted enzymatic "cascade" synthesis of PANI-ES (without volume inflation mechanism; **Fig. 4**). The plot is obtained from the 47 total observations; see also **Fig. 4b**.

b, Relative radii of daughter GUVs just after vesicle division when AOT micelles and SDBS + Chol mixed micelles were supplied to binary AOT + Chol (9/1) GUVs under the membrane-assisted enzymatic cascade synthesis of PANI-ES (with volume inflation mechanism; **Fig. 5**). It should be noted that the radius of each GUV was measured just after the division and that these radii increased further from the plotted values due to the osmotic swelling, as seen in **Fig. 5b**. The plot is obtained from the 50 total observations; see also **Fig. 5c**.

Supplementary Note 7.

Enthalpy change due to the interaction between AOT and PANI-ES.

In our synthetic minimal cell system, polyaniline in its emeraldine salt form, PANI-ES, encodes the vesicle-forming amphiphile with sulfonated head group (*e.g.,* AOT) in the linear *para*-NC sequence[4,7,12,20] due to the template effect[13,14] (**Fig. 2a**, **Fig. 2b**, and **Fig. 2d**). In contrast, the polymerisation reaction occurs randomly in the absence of template vesicles, resulting in the formation of a mixture of products which include significant amounts of branched compounds[12,42]. The heart of this template polymerisation mechanism lies in the specific interaction between vesicle-forming amphiphiles and PANI-ES. The bond energy between AOT and PANI-ES stabilises the linear *para*-NC sequence at the expense of the entropy loss due to the regular sequence (see **Discussion** section). In this section, we consider the enthalpy change (ΔH) due to the formation of PANI-ES–AOT complex, focusing on the specific hydrogen bonding between the sulfonate head group ($-SO_3^-$) of AOT and the cation radical amine ($>N^{\bullet+}-H$) of PANI-ES (**Supplementary Fig. 7**).

Without specific hydrogen bonding, the sulfonate head group and the positively charged amine radical are surrounded by water molecules (hydrated state; HS). According to a simulation study of an AOT membrane[43], on average, four water molecules are primarily bound to a sulfonate head group of an AOT molecule through hydrogen bonding. The bond energy between the head group and one water molecule[43] is calculated to be –7.39 kcal mol^{-1}, therefore, the enthalpy difference between a hydrated AOT head group, $-SO_3^-$(HS), and a naked state of $-SO_3^-$(NS), is estimated to be 29.6 kcal mol^{-1} (ΔH_1). The hydrogen bond energy between an amine group of PANI-ES and a water molecule was measured to be about –5.0 kcal mol^{-1} whether the amine group has a cation radical or not[44], and the value is supported by a simulation study[45]. This value corresponds to the enthalpy difference between a hydrated amine cation radical of PANI-ES, $-NH^{\bullet+}$ (HS), and a naked $-NH^{\bullet+}$ (NS) (ΔH_2). On the other hand, the amine radical cation group of PANI-ES directly interacts with the sulfonate head group through hydrogen bonding enhanced by electrostatic attraction, where the bond energy is estimated by a simulation study to be –65 kcal mol^{-1} (ΔH_3)[11]. This specific hydrogen bonding is significantly stronger if compared with typical hydrogen bonding[46,47] ranging from –4 to –12 kcal mol^{-1}. Such a strong attraction in the PANI-ES–AOT complex is supported by another simulation study[48]. Here, the enthalpy change from an isolated amine radical cation group of PANI-ES and an isolated sulfonate head group of AOT molecule to their complex is estimated to be –30.4 kcal mol^{-1} (ΔH_A), which corresponds to be –51.5 k_BT.

Thus, the PANI-ES chain is bound to the AOT vesicle surface through electrostatic attraction-involving hydrogen bonding between the amine radical cations of PANI-ES ($>N^{\bullet+}-H$) and the

sulfonate head group of AOT ($-SO_3^-$), as depicted in **Supplementary Fig. 1a**. It should be noted that such a "doped" PANI-ES tetraaniline repeating unit coupled with two counter ions has been widely accepted in studies of the doped, conductive form of polyaniline[49–51]. The estimated overall enthalpy change to form PANI-ES–AOT complexes is –60.8 kcal mol^{-1} (= –103 k_BT) per PANI-ES repeating unit. Although this is just a roughly estimated value, one can suppose that the enthalpy gain to form PANI-ES–AOT complexes is much larger than the free energy change due to the linear *para*-NC sequence ($-T\Delta S = 8\ln(2)\, k_BT = 5.5k_BT$ per PANI-ES repeating unit), which encourages the template polymerisation of aniline on AOT vesicles. This is the origin of the information encoding in our synthetic minimal cell system[52] (see **Discussion** section).

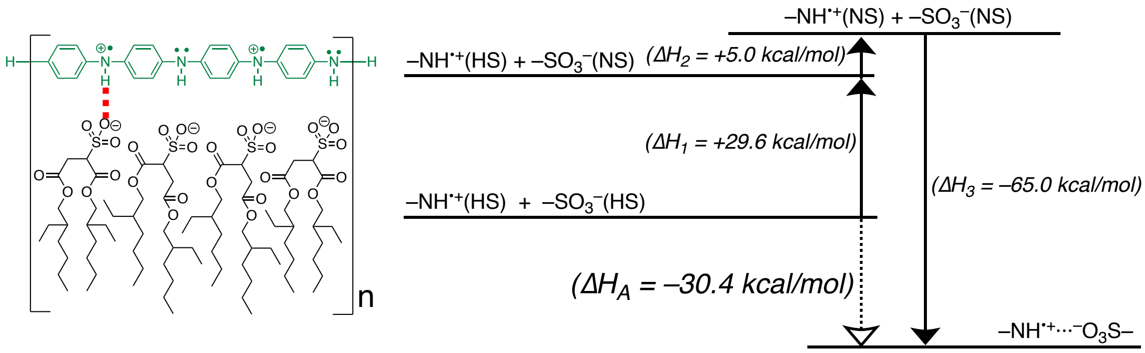

Supplementary Figure 7. Estimation of the enthalpy change by forming an electrostatic attraction-involving hydrogen bonding between the sulfonate head group of the AOT molecule and the amine radical cation group of PANI-ES.

Red dashed line in a chemical formula represents the bonding. "$-NH^{\bullet+}$"; amine radical cation group of PANI-ES, and "$-SO_3^-$"; sulfonate head group of AOT. "(HS)" represents the hydrated state, and "(NS)" represents the naked state. The black solid arrows represent estimated enthalpy changes in each step, and the open dashed arrow represents the estimated enthalpy difference between the hydrated state and the specific hydrogen bonding state.

Supplementary Note 8.

Deformation and division of vesicle induced by inverse cone-shaped amphiphile.

8-1. Theoretical analysis for the deformation of binary AOT + Chol vesicles.

We attained vesicle deformation to the limiting shape by introducing cholesterol (Chol) (**Fig. 4a** and **Fig. 5a**) or 1,2-dilauroyl-*sn*-glycero-3-phosphoethanolamine (DLPE) (**Supplementary Note 8-2**), which has negative molecular spontaneous curvature, into a GUV composed of AOT, which has a zero molecular spontaneous curvature. The deformation of AOT + Chol vesicle is described by the spontaneous curvature model[53,54] since AOT and Chol have fast flip-flop rates[19,55]. According to the spontaneous curvature model, the vesicle shape is determined by the reduced volume $v = V/[\left(\frac{4\pi}{3}\right) R_0^3]$ ($R_0 = \sqrt{A(t)/4\pi}$, V; vesicle volume and A; vesicle surface area) and the reduced spontaneous curvature $c_0 = C_0 R_0$ (C_0; spontaneous curvature)[56]. According to the phase diagram based on the spontaneous curvature model[56], the symmetric limiting shape vesicle is obtained at v = 0.7 and c_0 = 3. When c_0 is larger than 3, the vesicle deforms to the asymmetric limiting shape (formation of large and small daughter vesicles). On the contrary, when c_0 is smaller than 3, the vesicle cannot deform into the limiting shape. The spontaneous curvature for AOT + Chol binary vesicle is expressed by $C_0 = \frac{1}{2} H_{\mathrm{Chol}} \Delta\phi$, where H_{Chol} is the molecular spontaneous curvature of Chol (Chol is assumed to have the same molecular spontaneous curvature as DLPE, *i.e.*, $H_{\mathrm{Chol}} \sim -0.3$ nm^{-1} [57,58]) and $\Delta\phi = \phi^+ - \phi^-$ (ϕ^+ and ϕ^- are the area fraction of Chol in the outer and inner leaflet, respectively) is the area fraction difference of Chol between the outer and inner leaflet. For equilibrated bilayers, $\Delta\phi$ is determined by the equality of chemical potentials of Chol in the outer and inner leaflet, since Chol has a fast flip-flop rate[55]. The free energy of Chol in the outer leaflet of a spherical vesicle with radius R and membrane thickness h is expressed by

$$F_{\mathrm{Chol}}^{+} = \frac{\kappa}{2}\left(\frac{2}{R+\frac{h}{2}} - H_{\mathrm{Chol}}\phi^{+}\right)^{2} A^{+} + k_{\mathrm{B}}T\left(N_{\mathrm{AOT}}^{+} \ln(1-\phi^{+}) + N_{\mathrm{Chol}}^{+} \ln\phi^{+}\right), \quad \text{(S8-1)}$$

$$N^{+} = N_{\mathrm{AOT}}^{+} + N_{\mathrm{Chol}}^{+}, \quad \text{(S8-2)}$$

$$\phi^{+} = \frac{N_{\mathrm{Chol}}^{+}}{N^{+}}, \quad \text{(S8-3)}$$

where κ is the bending rigidity of binary vesicle composed of AOT and Chol, $A^{+} = aN^{+}$ (we assume that AOT and Chol have the same cross-section area a) is the membrane area of the outer

leaflet, N_{AOT}^{+} and N_{Chol}^{+} are numbers of AOT and Chol molecules in the outer leaflet, respectively. The chemical potential of Chol in the outer leaflet is obtained by

$$\mu_{\mathrm{Chol}}^{+} = \left(\frac{\partial F_{\mathrm{Chol}}^{+}}{\partial N_{\mathrm{Chol}}^{+}}\right)_{N_{\mathrm{AOT}}^{+}}$$

$$= -\kappa a H_{\mathrm{Chol}}\left(\frac{2}{R+\frac{h}{2}} - H_{\mathrm{Chol}}\phi^{+}\right)(1-\phi^{+}) + \frac{\kappa a}{2}\left(\frac{2}{R+\frac{h}{2}} - H_{\mathrm{Chol}}\phi^{+}\right)^{2} + k_{\mathrm{B}}T\ \ln\phi^{+} \qquad \text{(S8-4)}$$

Similarly, the chemical potential for Chol in the inner leaflet is obtained by

$$\mu_{\mathrm{Chol}}^{-} = \left(\frac{\partial F_{\mathrm{Chol}}^{-}}{\partial N_{\mathrm{Chol}}^{-}}\right)_{N_{\mathrm{AOT}}^{-}}$$

$$= \kappa a H_{\mathrm{Chol}}\left(\frac{2}{R+\frac{h}{2}} + H_{\mathrm{Chol}}\phi^{-}\right)(1-\phi^{-}) + \frac{\kappa a}{2}\left(\frac{2}{R+\frac{h}{2}} + H_{\mathrm{Chol}}\phi^{-}\right)^{2} + k_{B}T\ \ln\phi^{-} \qquad \text{(S8-5)}$$

From the equality of the chemical potential of Chol in the outer leaflet and inner leaflet, we obtain $\Delta\phi \sim -1.6 \times 10^{-5}$ for equilibrated binary AOT + Chol vesicle with $R = 10$ μm and $\bar{\phi} = \frac{1}{2}(\phi^{+} + \phi^{-}) = 0.1$, *i.e.*, the reduced spontaneous curvature of $c_0 = \frac{1}{2}H_{\mathrm{Chol}}\Delta\phi R_0 \sim 2.4 \cdot 10^{-2}$. Thus, an AOT + Chol vesicle cannot transform to the limiting shape vesicle as it is[59]. However, when AOT molecules in the external solution are incorporated into the outer leaflet of the vesicle bilayer through PANI-ES and then move to the inner leaflet by flip-flop motions, the concentration of Chol in the outer leaflet is determined by a balance of uptake rate and flip-flop rate of AOT. If the AOT uptake rate is faster than the AOT flip-flop rate, the concentration of Chol in the outer leaflet is diluted, which results in an increase of $\Delta\phi$. The $\Delta\phi$ required to attain the deformation to the symmetric limiting shape, *e.g.*, $c_0 \sim 3$, is only $\Delta\phi \sim -2 \cdot 10^{-3}$ for $R = 10$ μm and $\bar{\phi} = 0.1$. Thus, the AOT uptake rate determined by PANI-ES might be responsible for the deformation of AOT + Chol/PANI-ES vesicle to the limiting shape vesicle.

8-2. Growth and division of binary AOT + phosphatidylethanolamine (PE) vesicles.

A key to attain vesicle deformation to the limiting shape and spontaneous division is the coupling between the membrane curvatures and the molecular shape of the amphiphiles[59,60], as explained in the **Discussion** section.

Based on our previous study on the membrane growth and division of AOT + Chol (9/1) GUVs[20], we developed a recursive membrane growth/division (**Fig. 4a**) and, in the end, a synthetic minimal cell system (**Fig. 5a**) coupled with artificial metabolic pathways (**Fig. 1b**). In order to examine the effect of inverse-cone shaped amphiphile on vesicle growth and division, for reference to Chol, here we introduce DLPE, which is also a membrane component with a negative molecular spontaneous curvature of $H_{\mathrm{DLPE}} \sim -0.3$ nm^{-1} [57], into GUVs composed of

AOT, which has zero molecular spontaneous curvature.

The membrane growth and subsequent deformation of AOT + DLPE (9/1) GUVs coupled with the surface-confined PANI-ES synthesis were observed by the micro-injection experiments without the volume inflation mechanism. The AOT + DLPE (9/1) GUVs were prepared by the gentle hydration method in 20 mM NaH_2PO_4 solution (pH = 4.3), which was almost the same procedure as applied for the AOT + Chol GUVs (see **Methods**), except for the lipid used. Then, 2.0 mL of the AOT + DLPE GUV suspension mixed with the HRPC-catalysed polymerisation components, except for H_2O_2 to trigger the reaction, was carefully transferred at room temperature ($T \sim 25^{\circ}C$) from the Eppendorf tube into the sample chamber, a glass-bottom dish D11130H (Matsunami, Japan). The initial concentrations of each reaction component were as follows: 3.0 mM amphiphiles (GUVs; AOT/DLPE = 9/1), 4.0 mM aniline and 0.92 μM HRPC in 20 mM NaH_2PO_4 solution (pH = 4.3). Then, the polymerisation reaction was triggered by micro-injecting a 2.0 M H_2O_2 solution containing 20 mM AOT micelles, which was freshly prepared and pressed through a 0.2 μm polypropylene filter before use. During the micro-injection the GUVs were observed by phase-contrast microscopy (see **Methods**).

The AOT + DLPE (9/1) GUVs showed membrane growth and spontaneous division in response to the PANI-ES synthesis on their surface and incorporation of AOT molecules (**Supplementary Fig. 8**). The mother GUV started showing membrane growth ~15 sec after starting the micro-injection, and then did not deform to the prolate shape but deformed to the limiting shape at ~23 sec. Finally, the AOT + DLPE (9/1) GUV showed spontaneous division and produced two daughter GUVs at ~33 sec. This deformation pathway is very similar to that of AOT + Chol (9/1) GUVs (**Fig. 4a**), which supports the importance of introducing the second membrane component with an inverse-cone shape such as Chol for the vesicle reproduction due to the coupling of membrane curvature and local lipid composition of the binary vesicle (see **Discussion**).

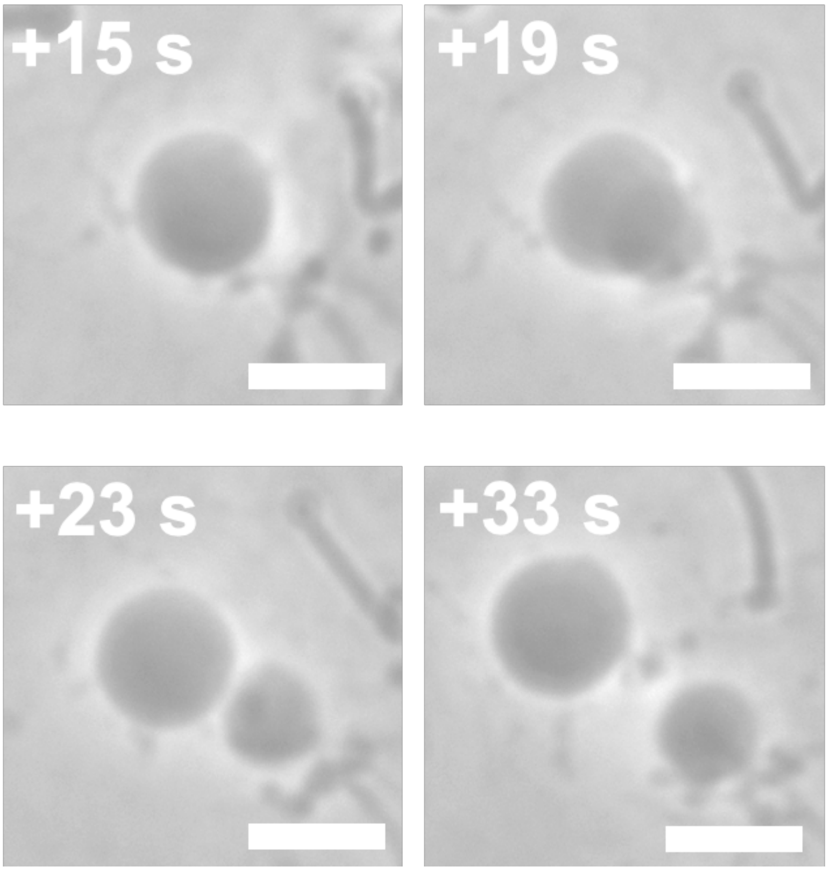

Supplementary Figure 8. Membrane growth and division of a binary AOT + DLPE GUV assisted by enzymatically formed PANI-ES.

Phase contrast light microscopy images of binary AOT + DLPE (9/1) GUV during the polymerisation reaction of aniline induced by the micro-injection of H_2O_2 and AOT micelles. The initial mother binary AOT+DLPE (9/1, 3.0 mM in total) GUV was prepared in 20 mM NaH_2PO_4 solution (pH = 4.3) containing 4.0 mM aniline and 0.92 μM HRPC. The freshly prepared 20 mM AOT micellar solution containing 2.0 M H_2O_2 was micro-injected to the GUV. The elapsed time after starting the micro-injection is indicated in each image. Length of the scale bars: 10 μm.

Supplementary Note 9.

Descriptions of the supporting movies.

Supplementary Movie 1. Original movie file for Fig. 3a.

Phase contrast light microscopy movie of the membrane growth of an AOT GUV promoted by enzymatic cascade synthesis of PANI-ES on the vesicle surface, triggered by the micro-injection of *AOT* and *D-glucose*. A 20 mM AOT micellar solution containing 100 mM D-glucose was micro-injected to the AOT GUV suspension containing 3.0 mM AOT, 4.0 mM aniline, 0.92 μM HRPC, 1.0 μM GOD, and dissolved oxygen in 20 mM NaH_2PO_4 solution (pH = 4.3) (see **Methods**). The targeted spherical AOT GUV showed membrane growth to a prolate shape by incorporating AOT molecules from the external solution, which was catalysed by the PANI-ES on the vesicle surface. Selected snapshots are shown in **Fig. 3a**. The elapsed time after starting the micro-injection of the AOT micellar solution and D-glucose is indicated at the upper left of the movie. Length of the scale bar: 10 μm.

Supplementary Movie 2. Original movie file for Fig. 4a.

Phase contrast light microscopy movie of the membrane growth and division of binary AOT + Chol (9/1) GUVs promoted by the enzymatic cascade synthesis of PANI-ES triggered by the micro-injection of *AOT*, *SDBS*, *Chol*, and *D-glucose* (*without* volume inflation mechanism). 100 mM D-glucose and micelles (AOT micelles and mixed SDBS + Chol (100/0.5) micelles) were micro-injected to a binary AOT + Chol GUV suspension containing 3.0 mM amphiphiles (AOT and Chol), 4.0 mM aniline, 0.92 μM HRPC, 1.0 μM GOD, dissolved oxygen, and 100 mM D-sucrose in 20 mM NaH_2PO_4 solution (pH = 4.3) (see **Methods**). The targeted spherical binary AOT + Chol (9/1) GUV showed membrane growth and deformation to the limiting shape instead of the prolate shape observed in the experiments with AOT GUVs. The neck was then destabilised, *i.e.*, the GUV showed division. This membrane growth and division cycle of GUVs were observed over several generations. Selected snapshots are shown in **Fig. 4a**. The elapsed time after starting the micro-injection is indicated in the upper left of the movie. Length of the scale bar: 10 μm.

Supplementary Movie 3. Original movie file for Fig. 5a.

Phase contrast light microscopy movie of the recursive reproduction of binary AOT + Chol (9/1) GUVs promoted by the enzymatic cascade synthesis of PANI-ES triggered by the micro-injection of *AOT*, *SDBS*, *Chol*, and *D-glucose* (*with* volume inflation mechanism), *i.e.*, our synthetic minimal cell system. A binary AOT + Chol (9/1) GUV was transferred from the reaction mixture prepared for the HRPC/GOD-catalysed polymerisation of aniline containing *100 mM D-*

sucrose into the same reaction mixture but containing *100 mM D-fructose*. Then, 100 mM D-glucose and micelles (AOT micelles and mixed SDBS + Chol (100/0.5) micelles) were micro-injected to the targeted binary AOT + Chol GUV to trigger the polymerisation reaction and the subsequent membrane growth (see **Methods**). The targeted initially spherical binary AOT + Chol (9/1) GUV showed membrane growth and division until the micro-injection ceased at ~70 sec. Then, the GUV kept its spherical shape (while a few small GUVs were budded off from the existing GUV) and increased their volume by the osmotic swelling. Finally, two out of three daughter GUVs under the micro-injection became the same or a larger size than their originating initial mother GUV. Selected snapshots are shown in **Fig. 5a**. The elapsed time after starting the micro-injection is indicated in the upper left of the movie. Length of the scale bar: 10 μm.

Supplementary Movie 4, 5, and 6. Additional movies of recursive vesicle reproduction.

Additional phase contrast light microscopy movies of the recursive reproduction of binary AOT + Chol (9/1) GUVs coupled with the enzymatic synthesis of PANI-ES, which was observed in the same experimental system as in the experiments shown in **Supplementary Movie 3**. The elapsed time after starting the micro-injection is indicated in the upper left of each movie. Length of the scale bar: 10 μm. **Movie 4**: The micro-injection was manually applied twice to the targeted binary AOT + Chol (9/1) GUVs, and the GUVs showed ON/OFF of the membrane growth/division cycle. The first micro-injection at ~0–30 sec induced the vesicle growth/ division cycle twice. Then the micro-injection ceased, and the second micro-injection at ~95–115 sec again induced the vesicle growth and division cycles, while the difference in the size of daughter GUVs was enlarged. **Movie 5**: The micro-injection was continuously applied to the targeted binary AOT + Chol (9/1) GUVs. The targeted spherical binary AOT + Chol (9/1) GUV showed twice successive growth and division cycles at ~20 sec, and then relatively smaller GUVs were produced at ~47 sec. **Movie 6**: Three binary AOT + Chol (9/1) GUVs were targeted, and all of them showed reproduction at the same time. The micro-injection was first applied to the two GUVs at ~0 sec. The upper one (indicated by a white arrow) showed the first growth and division at ~33 sec. Then, the micro-injection was applied to one of the daughter GUVs and the other two GUVs at ~38 sec, and then all three GUVs completed growth and division cycles at almost the same time at ~58 sec. These movies demonstrate the reproducibility of our experiments about the recursive reproduction of the synthetic minimal cells developed in this work.

Supplementary Note 10.

Supplementary References

1. Bankar, S. B., Bule, M. V., Singhal, R. S. & Ananthanarayan, L. Glucose oxidase - An overview. *Biotechnol. Adv.* **27**, 489–501 (2009).
2. Genies, E. M. & Tsintavis, C. Redox mechanism and electrochemical polyaniline deposits behaviour of polyaniline deposits. *J. Electroanal. Chem.* **195**, 109–128 (1985).
3. Ding, Y., Padias, A.B., & Hall, H.K. Chemical Trapping Experiments Support a Cation-Radical Mechanism for the Oxidative Polymerization of Aniline. *J. Polym. Sci. Part A: Polym. Chem.* **37**, 2569-1579 (1999)
4. Junker, K. et al. Mechanistic aspects of the horseradish peroxidase-catalysed polymerisation of aniline in the presence of AOT vesicles as templates. *RSC Adv.* **2**, 6478–6495 (2012).
5. Guo, Z. et al. Vesicles as soft templates for the enzymatic polymerization of aniline. *Langmuir* **25**, 11390–11405 (2009).
6. Guo, Z., Hauser, N., Moreno, A., Ishikawa, T. & Walde, P. AOT vesicles as templates for the horseradish peroxidase-triggered polymerization of aniline. *Soft Matter* **7**, 180–193 (2011).
7. Junker, K., Zandomeneghi, G., Schuler, L. D., Kissner, R. & Walde, P. Enzymatic polymerization of pyrrole with Trametes versicolor laccase and dioxygen in the presence of vesicles formed from AOT (sodium bis-(2-ethylhexyl) sulfosuccinate) as templates. *Synth. Met.* **200**, 123–134 (2015).
8. Iwasaki, F., Luginbühl, S., Suga, K., Walde, P. & Umakoshi, H. Fluorescent Probe Study of AOT Vesicle Membranes and Their Alteration upon Addition of Aniline or the Aniline Dimer *p*-Aminodiphenylamine (PADPA). *Langmuir* **33**, 1984–1994 (2017).
9. Dunford, H. B., Hewson, W. D. & Steiner, H. Horseradish peroxidase. XXIX. Reactions in water and deuterium oxide: cyanide binding, compound I formation, and reactions of compounds I and II with ferrocyanide. *Can. J. Chem.* **56**, 2844–2852 (1978).
10. Junker, K., Gitsov, I., Quade, N. & Walde, P. Preparation of aqueous polyaniline-vesicle suspensions with class III peroxidases. Comparison between horseradish peroxidase isoenzyme C and soybean peroxidase. *Chem. Pap.* **67**, 1028–1047 (2013).
11. Casanovas, J., Canales, M., Ferreira, C. A. & Alema, C. A. First principle analysis of the structure of oligoanilines doped with alkylsulfonic acids. *J. Phys. Chem. A* **113**, 8795–8800 (2009).
12. Luginbühl, S. et al. The influence of anionic vesicles on the oligomerization of *p*-aminodiphenylamine catalyzed by horseradish peroxidase and hydrogen peroxide. *Synth. Met.* **226**, 89–103 (2017).

13. Połowiński, S. Template polymerisation and co-polymerisation. *Prog. Polym. Sci.* **27**, 537–577 (2002).

14. Serrano-Luginbühl, S., Ruiz-Mirazo, K., Ostaszewski, R., Gallou, F. & Walde, P. Soft and dispersed interface-rich aqueous systems that promote and guide chemical reactions. *Nat. Rev. Chem.* **2**, 306–327 (2018).

15. Kurisu, M., Kissner, R., Imai, M. & Walde, P. Application of an enzymatic cascade reaction for the synthesis of the emeraldine salt form of polyaniline. *Chem. Pap.* **75**, 5071–5085 (2021).

16. Foreman, J. P. & Monkman, A. P. Theoretical investigations into the structural and electronic influences on the hydrogen bonding in doped polyaniline. *Synth. Met*. **107**, 7604–7610 (2003).

17. Junker, K. et al. Efficient polymerization of the aniline dimer *p*-aminodiphenylamine (PADPA) with *Trametes versicolor* laccase/O2 as catalyst and oxidant and AOT vesicles as templates. *ACS Catal.* **4**, 3421–3434 (2014).

18. Kashima, K. et al. How experimental details matter. The case of a laccase-catalysed oligomerisation reaction. *RSC Adv.* **8**, 33229–33242 (2018).

19. Poghosyan, A.H. & Mamasakhlisov, Y.Sh. The mechanism of flip-flops in a AOT lamella: A molecular dynamics study. *Colloids Surf. A* **642**, 128681 (2022).

20. Kurisu, M. et al. Reproduction of vesicles coupled with a vesicle surface-confined enzymatic polymerisation. *Commun. Chem.* **2**:117 (2019).

21. Huang, W. S. & MacDiarmid, A. G. Optical properties of polyaniline. *Polymer.* **34**, 1833–1845 (1993).

22. Nekrasov, A. A., Ivanov, V. F. & Vannikov, A. V. Effect of pH on the structure of absorption spectra of highly protonated polyaniline analyzed by the Alentsev-Fock method. *Electrochim. Acta* **46**, 4051–4056 (2001).

23. do Nascimento, G. M. & de Souza, M. A. (2010). Eftekhari, A. (ed) Spectroscopy of Nanostructured Conducting Polymers. In: Nanostructured conductive polymers. *Wiley, Chichester*.

24. Bilal, S., Gul, S., Holze, R. & Shah, A. U. H. A. An impressive emulsion polymerization route for the synthesis of highly soluble and conducting polyaniline salts. *Synth. Met.* **206**, 131–144 (2015).

25. Koonin, E.V. How many genes can make a cell: the minimal-gene-set concept. *Annu. Rev. Genom. Hum. Genet.* **1**, 99 (2000).

26. Xu, P. et al. Genome-wide essential gene identification in *Streptococcus sanguinis*. *Sci. Rep.* **1**, 1–9 (2011).

27. Bright, H. J. & Porter, D. J. T. (1975). Boyer, P.D. (Ed.), Flavoprotein oxidases, In: The Enzymes vol. 12 (3rd ed.) *NewYork: Academic Press.*

28. Leskovac, V., Trivić, S., Wohlfahrt, G., Kandrač, J. & Peričin, D. Glucose oxidase from *Aspergillus niger*: The mechanism of action with molecular oxygen, quinones, and one-electron acceptors. *Int. J. Biochem. Cell Biol.* **37**, 731–750 (2005).
29. Qin, L., Tripathi, G. N. R. & Schüler, R. H. Radiation Chemical Studies of the Oxidation of Aniline in Aqueous Solution. *Z. Naturforsch.* **40**, 1026–1039 (1985).
30. Dunford, H.B. (1999) *Heme Peroxidases.* John Wiley & Sons.
31. Sapurina, I., Riede, A. & Stejskal, J. In-situ polymerized polyaniline films - 3. Film formation. *Synth. Met.* **123**, 503–507 (2001).
32. Weibel, M. K. & Bright, H. J. The glucose oxidase mechanism. Interpretation of the pH dependence. *J. Biol. Chem.* **246**, 2734–2744 (1971).
33. Araiso, T. & Dunford, H. B. Horseradish peroxidase. XLI. Complex formation with nitrate and its effect upon compound I formation. *J. Chem. Inf. Model.* **53**, 1689–1699 (1980).
34. Job, D. & Dundord, H. B. Substituent Effect on the Oxidation of Phenols and Aromatic Amines by Horseradish Peroxidase Compound I. *Eur. J. Biochem.* **66**, 607–614 (1976).
35. Sakurada, J., Sekiguchi, R., Sato, K. & Hosoya, T. Kinetic and Molecular Orbital Studies on the Rate of Oxidation of Monosubstituted Phenols and Anilines by Horseradish Peroxidase Compound II. *J. Biol. Phys.* **29**, 4093–4098 (1990).
36. Reynafarje, B., Costa, L.E., & Lehninger, A.L. O2 solubility in aqueous media determined by a kinetic method. *Anal. Biochem.* **145**, 406-418 (1985).
37. Millero, F.J., Huang, F., & Laferiere, A.L. The solubility of oxygen in the major sea salts and their mixtures at 25°C. *Geochim. Cosmochim. Acta* **66**, 2349-2359 (2002).
38. Toyobo Enzymes, Product Information.
https://www.toyobo-global.com/seihin/xr/enzyme/pdf_files/201707/PEO_131_301_302.pdf
39. Grillo, I., Levitz, P. & Zemb, T. Insertion of small anionic particles in negatively charged lamellar phases. *Langmuir* **16**, 4830–4839 (2000).
40. Mally, M., Peterlin, P. & Svetina, S. Partitioning of oleic acid into phosphatidylcholine membranes is amplified by strain. *J. Phys. Chem. B* **117**, 12086–12094 (2013).
41. Schindelin, J. et al. Fiji: An open-source platform for biological-image analysis. *Nat. Methods* **9**, 676–682 (2012).
42. Luginbühl, S., Bertschi, L., Willeke, M., Schuler, L. D. & Walde, P. How anionic vesicles steer the oligomerization of enzymatically oxidized *p*-aminodiphenylamine (PADPA) toward a polyaniline emeraldine salt (PANI-ES)-type product. *Langmuir* **32**, 9765–9779 (2016).
43. Chanda, J., Chakraborty, S. & Bandyopadhyay, S. Monolayer of aerosol-OT surfactants adsorbed at the air/water interface: An atomistic computer simulation study. *J. Phys. Chem. B* **109**, 471–479 (2005).

44. Matveeva, E. S., Diaz Calleja, R. & Parkhutik, V. P. Thermogravimetric and calorimetric studies of water absorbed in polyaniline. *Synth. Met.* **72**, 105–110 (1995).

45. Ostwal, M. M., Sahimi, M. & Tsotsis, T. T. Water harvesting using a conducting polymer: A study by molecular dynamics simulation. *Phys. Rev. E* **79**, 1–16 (2009).

46. Alemán, C. On the ability of modified peptide links to form hydrogen bonds. *J. Phys. Chem. A* **105**, 6717–6723 (2001).

47. Colominas, C., Teixidó, J., Cemelí, J., Luque, F. J. & Orozco, M. Dimerization of carboxylic acids: Reliability of theoretical calculations and the effect of solvent. *J. Phys. Chem. B* **102**, 2269–2276 (1998).

48. Vaschetto, M. E., Retamal, B. A., Contreras, M. L. & Zagal, J. H. Theoretical analysis of counterfoil influence over the physicochemical properties of aniline tetramers in several oxidation states. *Struct. Chem.* **8**, 121–129 (1997).

49. Teasdale, P. R. & Wallace, G. G. Molecular recognition using conducting polymers: basis of an electrochemical sensing technology. *Analyst* **4**, 329–334 (1993).

50. Walde, P. & Guo, Z. Enzyme-catalyzed chemical structure-controlling template polymerization. *Soft Matter* **7**, 316–331 (2011).

51. Jaymand, M. Recent progress in chemical modification of polyaniline. *Prog. Polym. Sci.* **38**, 1287–1306 (2013).

52. Schneider, T. D. A brief review of molecular information theory. *Nano Commun. Netw.* **1**, 173–180 (2010).

53. Helfrich, W. Elastic Properties of Lipid Bilayers: Theory and Possible Experiments. *Z. Naturforsch. C* **28**, 693–703 (1973).

54. Urakami, N., Sakuma, Y., Chiba, T. & Imai, M. Vesicle deformation and division induced by flip-flops of lipid molecules. *Soft Matter* **17**, 8434–8445 (2021).

55. Hamilton, J. A. Fast flip-flop of cholesterol and fatty acids in membranes: implications for membrane transport proteins. *Curr. Opin. Lipidol.* **14**, 263–271 (2003).

56. Seifert, U., Berndl, K. & Lipowsky, R. Shape transformations of vesicles: Phase diagram for spontaneous curvature and bilayer-coupling models. *Phys. Rev. A* **44**, 1182–1202 (1991).

57. Kamal, M. M., Mills, D., Grzybek, M. & Howard, J. Measurement of the membrane curvature preference of phospholipids reveals only weak coupling between lipid shape and leaflet curvature. *Proc. Natl. Acad. Sci. U. S. A.* **106**, 22245–22250 (2009).

58.Zimmerberg, J. & Kozlov, M. M. How proteins produce cellular membrane curvature. *Nat. Rev. Mol. Cell Biol.* **7**, 9–19 (2006).

59. Imai, M., Sakuma, Y., Kurisu, M. & Walde, P. From vesicles toward protocells and minimal cells. *Soft Matter* **18**, 4823–4849 (2022).

60. Sakuma,Y. & Imai,M. Model system of self-reproducing vesicles. *Phys.Rev.Lett.***107**,1–5 (2011).
