# Supplementary material for: Synthesising a minimal cell with artificial metabolic pathways
Source: Commun Chem. 2023 Mar 28;6:56. doi: 10.1038/s42004-023-00856-y (PMC10050237; doi:10.1038/s42004-023-00856-y)
Supplement: Supplementary file 3 — Description of Additional Supplementary Files [file 42004_2023_856_MOESM3_ESM.pdf]

Description of Additional Supplementary Files

File name: Supplementary Movie 1

Description: Original movie file for Fig. 3a.

File name: Supplementary Movie 2

Description: Original movie file for Fig. 4a.

File name: Supplementary Movie 3

Description: Original movie file for Fig. 5a.

File name: Supplementary Movie 4

Description: Additional movies of recursive vesicle reproduction.

File name: Supplementary Movie 5

Description: Additional movies of recursive vesicle reproduction.

File name: Supplementary Movie 6

Description: Additional movies of recursive vesicle reproduction.
